# In silico dynamics of COVID-19 phenotypes for optimizing clinical management

**DOI:** 10.1073/pnas.2021642118

**Published:** 2021-01-05

**Authors:** Chrysovalantis Voutouri, Mohammad Reza Nikmaneshi, C. Corey Hardin, Ankit B. Patel, Ashish Verma, Melin J. Khandekar, Sayon Dutta, Triantafyllos Stylianopoulos, Lance L. Munn, Rakesh K. Jain

**Affiliations:** ^a^Cancer Biophysics Laboratory, Department of Mechanical and Manufacturing Engineering, University of Cyprus, 1678 Nicosia, Cyprus;; ^b^Department of Mechanical Engineering, Sharif University of Technology, Tehran, Iran, 11155;; ^c^Edwin L. Steele Laboratories, Department of Radiation Oncology, Massachusetts General Hospital and Harvard Medical School, Boston, MA 02114;; ^d^Department of Pulmonary and Critical Care Medicine, Massachusetts General Hospital and Harvard Medical School, Boston, MA 02114;; ^e^Department of Medicine/Renal Division, Brigham and Women’s Hospital and Harvard Medical School, Boston, MA 02115;; ^f^Department of Radiation Oncology, Massachusetts General Hospital and Harvard Medical School, Boston, MA 02114;; ^g^Department of Emergency Medicine, Massachusetts General Hospital and Harvard Medical School, Boston, MA 02114

**Keywords:** SARS-CoV-2, COVID-19, mathematical model, simulation

## Abstract

Understanding the underlying mechanisms of COVID-19 progression and the impact of various pharmaceutical interventions is crucial for the clinical management of the disease. We developed a comprehensive mathematical framework based on the known mechanisms of the severe acute respiratory syndrome coronavirus 2 (SARS-CoV-2) infection, incorporating the renin−angiotensin system and ACE2, which the virus exploits for cellular entry, key elements of the innate and adaptive immune responses, the role of inflammatory cytokines, and the coagulation cascade for thrombus formation. The model predicts the evolution of viral load, immune cells, cytokines, thrombosis, and oxygen saturation based on patient baseline condition and the presence of comorbidities. Model predictions were validated with clinical data from healthy people and COVID-19 patients, and the results were used to gain insight into identified risk factors of disease progression including older age; comorbidities such as obesity, diabetes, and hypertension; and dysregulated immune response. We then simulated treatment with various drug classes to identify optimal therapeutic protocols. We found that the outcome of any treatment depends on the sustained response rate of activated CD8^+^ T cells and sufficient control of the innate immune response. Furthermore, the best treatment—or combination of treatments—depends on the preinfection health status of the patient. Our mathematical framework provides important insight into SARS-CoV-2 pathogenesis and could be used as the basis for personalized, optimal management of COVID-19.

## Significance

A distinctive feature of COVID-19 is its extreme heterogeneity—illness ranges from minimally symptomatic to life threatening. Heterogeneity results from a poorly understood combination of patient factors, viral dynamics, antiviral and immune modulating therapies, and dynamics of the innate and adaptive immune responses. In order to better understand clinical heterogeneity and optimal treatment, we developed a comprehensive mathematical model incorporating elements of the innate and adaptive immune responses, the renin−angiotensin system (which the virus exploits for cellular entry), rates of viral replication, inflammatory cytokines, and the coagulation cascade. Our model reveals divergent treatment responses and clinical outcomes as a function of comorbidities, age, and details of the innate and adaptive immune responses which can provide a framework for understanding individual patients’ trajectories.

COVID-19 has created unprecedented challenges for the health care system, and, until an effective vaccine is developed and made widely available, treatment options are limited. A challenge to the development of optimal treatment strategies is the extreme heterogeneity of presentation. Infection with severe acute respiratory syndrome coronavirus 2 (SARS-CoV-2) results in a syndrome that ranges in severity from asymptomatic to multiorgan failure and death. In addition to local complications in the lung, the virus can cause systemic inflammation and disseminated microthrombosis, which can cause stroke, myocardial infarction, or pulmonary emboli (1–4). Risk factors for poor COVID-19 outcome include advanced age, obesity, diabetes, and hypertension (5–13).

Computational analyses can provide insights into the transmission, control, progression, and underlying mechanisms of infectious diseases. Indeed, epidemiological and statistical modeling has been used for COVID-19, providing powerful insights into comorbidities, transmission dynamics, and control of the disease (14–17). However, to date, these analyses have been population dynamics models of SARS-CoV-2 infection and transmission or correlative analyses of COVID-19 comorbidities and treatment response. Simple viral dynamics models have been also developed and used to predict the SARS-CoV-2 response to antiviral drugs (18, 19). These models, however, do not explicitly consider the biological or physiological mechanisms underlying disease progression or the time course of response to various therapeutic interventions, and only a few more-sophisticated models have been developed toward this direction (20, 21).

Several therapies targeting various aspects of COVID-19 pathogenesis have been proposed and have either completed—or are currently being tested in—clinical trials (22). Despite strong biologic rationale, these treatments have generally produced conflicting results in the clinic. For example, trials of antiviral therapies (e.g., remdesivir) have been mixed: The original trial from China failed (23), a subsequent trial in the United States led to approval of remdesivir in the United States and other countries (24), and the recent results of the World Health Organization Solidarity trial again show no benefit (25). Other antiviral drugs alone or in combination are also showing promise (26).

Other potential treatments include antiinflammatory drugs and antithrombotic agents. Because of the systemic inflammation seen in many patients, antiinflammatory drugs have been tested, including anti-IL6/IL6R therapy (e.g., tocilizumab, siltuximab) and anti-JAK1/2 drugs (e.g., barcitinib). It is not clear whether these drugs will be effective as stand-alone treatments, particularly after the recent failure of tocilizumab in a phase III trial (1, 27–29). In addition, given that a common complication of COVID-19 is the development of coagulopathies with microvascular thrombi potentially leading to the dysfunction of multiple organ systems (2, 3), antithrombotic drugs (e.g., low molecular weight heparin) are being tested. Recognizing the interactions of COVID-19 with the immune system (30), the corticosteroid dexamethasone has been tested, showing some promising results. Given the large range of patient comorbidities, disease severities, and variety of complications such as thrombosis, it is likely that patients will have heterogeneous responses to any given therapy, and such heterogeneity will continue to be a challenge for clinical trials of unselected COVID-19 patients (31).

**Fig. 1.**
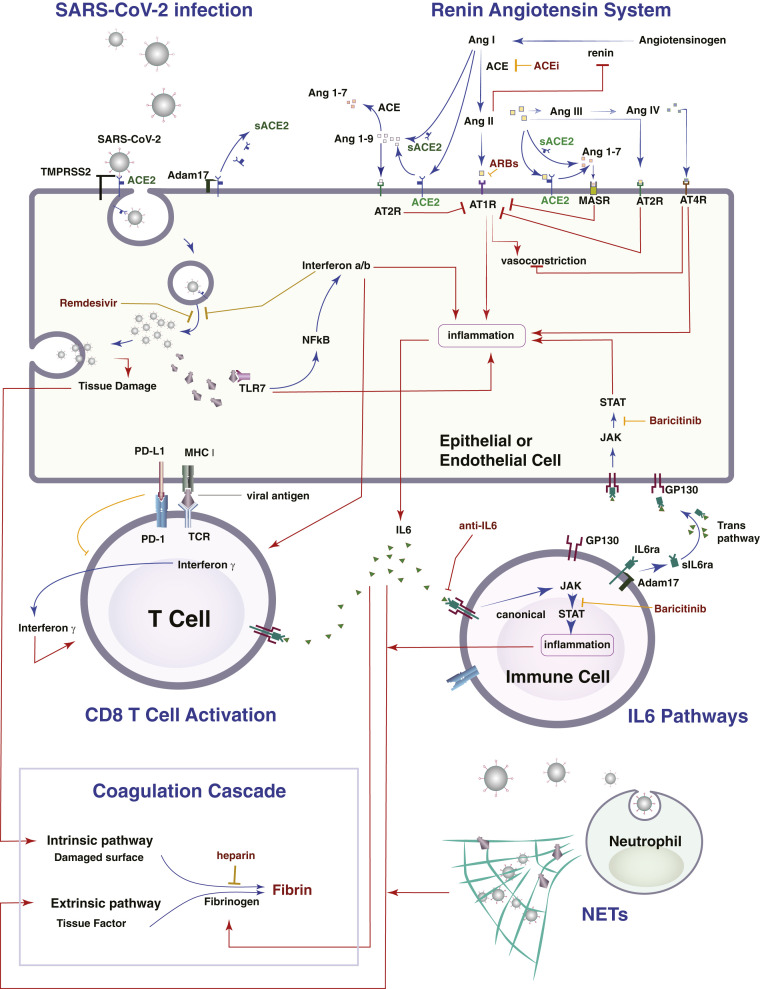
Schematic of the detailed lung model. The model incorporates the virus infection of epithelial and endothelial cells, the RAS, T cells activation and immune checkpoints, the known IL6 pathways, neutrophils, and macrophages, as well as the formation of NETs, and the coagulation cascade. The lung model is coupled with a PK/PD model for the virus and thrombi dissemination through the body.

Here, we developed a systems biology-based mathematical model to address this urgent need. Our model incorporates the known mechanisms of SARS-CoV-2 pathogenesis and the potential mechanisms of action of various therapeutic interventions that have been tested in COVID-19 patients. In previous work, we have exploited angiotensin receptor blockers (ARBs) and angiotensin converting enzyme inhibitors (ACEis) for the improvement of cancer therapies and developed mathematical models of the renin−angiotensin system in the context of cancer desmoplasia (32–35). Using a similar approach, we developed a detailed model that includes lung infection by the SARS-CoV-2 virus and a pharmacokinetic/pharmacodynamic (PK/PD) model of infection and thrombosis to simulate events that take place throughout the body during COVID-19 progression (Fig. 1 and *SI Appendix*, Fig. S1). The model is first validated against clinical data of healthy people and COVID-19 patients and then used to simulate disease progression in patients with specific comorbidities. Subsequently, we present model predictions for various therapies currently employed for treatment of COVID-19 alone or in combination, and we identify protocols for optimal clinical management for each of the clinically observed COVID-19 phenotypes.

## Model Description

The model includes SARS-CoV-2 infection, the renin angiotensin system (RAS), inflammatory and antiinflammatory cytokines, innate and adaptive immune cells, and factors involved in the coagulation cascade (Fig. 1). SARS-CoV-2 enters the cell by docking to ACE2, a key component of the RAS. ACE2 can be membrane bound or soluble, and it regulates inflammation by converting angiotensin (Ang) II to Ang 1–7 and Ang I to Ang 1–9; as opposed to Ang I and Ang II, which lead to inflammation. Ang 1–7 and Ang 1–9 have antiinflammatory effects. Intracellular virus initiates inflammatory pathways through toll-like receptors and NFκB, which produces interferons and other inflammatory cytokines. The viral antigens, along with inflammatory cytokines, cause activation of naïve T cells, creating virus-specific T effector cells. T cell activation is controlled by viral antigen strength and the presence of PD-L1/PD-1 inhibition (36). We combine inflammatory cytokines into a single variable, but explicitly account for IL6 production via the trans pathway in epithelial and endothelial cells and the canonical pathway in immune cells. In the presence of inflammatory cytokines and virus, neutrophils can produce neutrophil extracellular traps (NETs).

Because the virus can infect endothelial cells, we also consider viral dissemination via the blood stream, and the possibility of systemic infection and thrombosis. We include the major organs in a PK/PD model, with physiological blood flow patterns explicitly modeled. Infection of endothelial cells, combined with high levels of inflammatory cytokines in the plasma, can result in thrombosis. Damage to virally infected endothelial cells and the production of NETs can exacerbate the thrombosis, and microthrombi can enter the blood stream to accumulate in other organs, including the brain, heart, and lung. We use a simplified model of the coagulation pathways, assuming that formation of microthrombi is proportional to the number of infected endothelial cells, the presence of neutrophil NETs, and the level of inflammatory cytokines. Transport of oxygen from the alveolar space to the blood vessels in the lung is calculated using a modified diffusion model (37).

## Results

### Model Validation with Data of Healthy People and Various COVID-19 Phenotypes.

**Fig. 2.**
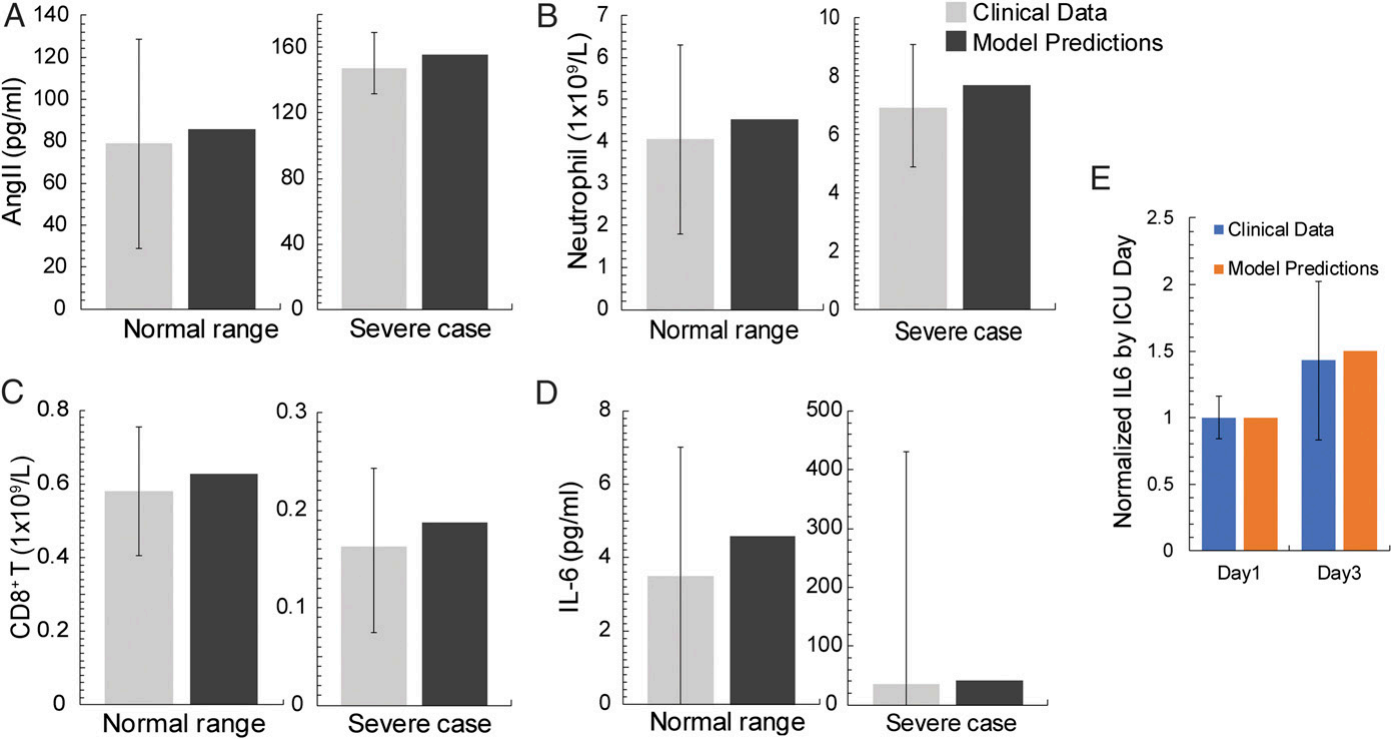
Validation of the model. (*A*–*D*) Model comparison with clinical data for healthy, uninfected people and for severe COVID-19 patients taken from pertinent studies (38–40). (*E*) Comparison of model predictions with IL6 clinical data from patients hospitalized at the Massachusetts General Hospital. The data were taken at day 1 and day 3 from the time of patient’s admission to the Intensive Care Unit (ICU).

Model predictions were validated with clinical data from healthy humans and severe COVID-19 patients (38–40). Fig. 2 *A*–*D* presents the comparison showing agreement between model predictions and clinical data for Ang II, neutrophils, CD8^+^ T cells, and IL6 levels. Fig. 2*E* further validates model predictions for the evolution of IL6 in COVID-19 patients admitted to the Intensive Care Unit of Massachusetts General Hospital. The use of retrospective patient data was deemed exempt by our institutional review board. Informed consent was waived (41).

**Fig. 3.**
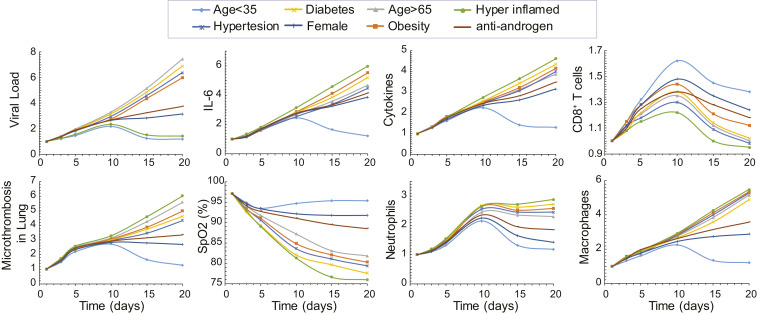
Dynamics of disease progression. Time evolution of key model variables: viral load, IL6 concentration, proinflammatory cytokines concentration, activated T cells, microthrombosis in the lung, saturated oxygen levels, neutrophils, and macrophages for young (<35 y old) and old (>65 y old) male patients, females (>65 y old), and old male patients with comorbidities: hypertension, obesity, and diabetes, as well as for hyperinflamed patients and for antiandrogen treatment. Values have been normalized to the corresponding initial values except for S(Po_2_). The response of a young female is similar to that of the young male and has been omitted from the plots.

Subsequently, we focused on the clinically observed phenotypes of COVID-19. We used the model to predict the time evolution of critical disease variables, namely, the virus load, levels of IL6 and other proinflammatory cytokines, the formation of microthrombi in the lung; the numbers of neutrophils, macrophages, and activated cytotoxic CD8^+^ T cells; and the blood oxygen saturation (SpO_2_). The COVID-19 patient phenotypes considered include young patients (age < 35 y), older patients (age > 65 y) who more frequently require hospitalization, and female patients, as well as patients with comorbidities such as hypertension, obesity, and diabetes, and patients with a dysregulated immune response, whose condition is characterized by high levels of proinflammatory cytokines (Fig. 3). *SI Appendix*, Table S2 presents the model parameters that were modified from the baseline values to account for these various patient phenotypes. To test the sensitivity of the model predictions, a parametric analysis of these model parameters was also performed by varying all parameters involved in each phenotype either together or separately (*SI Appendix*, Figs. S2–S4).

In all phenotypes, the viral load increases during early lung infection, with divergent trajectories seen after day 5, depending on the levels of activated T cells. In younger patients (age < 35 y) with a healthy immune system, a sustained recruitment of T cells is observed along with a reduction in viral load and inflammation, as well as a decrease in neutrophils and macrophages. All these effects cause a significant reduction in the formation of thrombi and restoration of oxygenation.

In general, the simulations resulting in poor outcome were due to increased baseline inflammation or more active innate immune response combined with less effective adaptive immunity. This was the case—in varying degrees—for older patients and those with diabetes, obesity, hypertension, and dysregulated immunity (“hyperinflamed”; see *SI Appendix*, Table S2 for the parameters varied in each population). In the latter case, the adaptive immune response is intact and clears the virus, but the innate immune response is sustained, perpetuating inflammation, thrombosis, and hypoxemia. Furthermore, the progression of the disease relates to the levels of initial viremia, and low levels of viral load can lead to virus clearance even for older, at-risk patients (*SI Appendix*, Fig. S5) (42).

An outstanding question is why males tend to have more severe COVID-19 disease compared with females (43). Proposed mechanisms include the higher CD4/CD8 T cell ratio in females (44) and androgen-induced differences in susceptibility to viral entry into the cells due to higher TMPRSS2 levels in males (45). To simulate COVID-19 in an older female, we decreased the production of naïve T cells and the rate of virus entry into the cells (46). The simulations predict lower viral load compared with the older males, with correspondingly less inflammation and hypoxemia. These results suggest that reduced viral entry into cells due to lower TMPRSS2 level can explain the improved outcomes in females—even if the adaptive immune response is not as vigorous (43).

### Efficacy of Current Treatments on COVID-19 Progression.

To identify optimal treatment protocols, we simulated the effects of the various treatments currently being investigated for COVID-19. We first simulated a patient of age >65 y (assumed to have baseline inflammation and impaired T effector cell function) and performed simulations of various treatments given at early (day 3, SpO2∼92%) and late (day 7, SpO2 < 90%) stages of the disease. For the purpose of the simulations, day 0 is the time of arrival of the virus in the lung, so significant symptoms are expected starting around day 3. Simulating treatment on day 3 represents early treatment (e.g., upon hospital admission); later treatment (day 7) could be due to delayed diagnosis/hospital admission or in response to disease progression. Treatments that were considered include heparin (anticoagulant), immune checkpoint inhibition (ICI), antiviral therapy, dexamethasone, ARBs, ACEi, hrACE2, anti-IL6, and anti-IL6R treatment. *SI Appendix*, Table S2 summarizes the model parameters that were modified from their baseline values to account for the different treatments. The severity of disease was primarily assessed based on the degree of hypoxemia and thrombus formation. The dynamics of the disease progression are shown in *SI Appendix*, Figs. S6–S9, and the values at the last day of the simulation (day 20) are summarized in Table 1.

**Table 1.** Summary of model predictions for the therapeutic outcome of currently employed treatments initiated either in the beginning (day 3) of the disease or later (day 7)

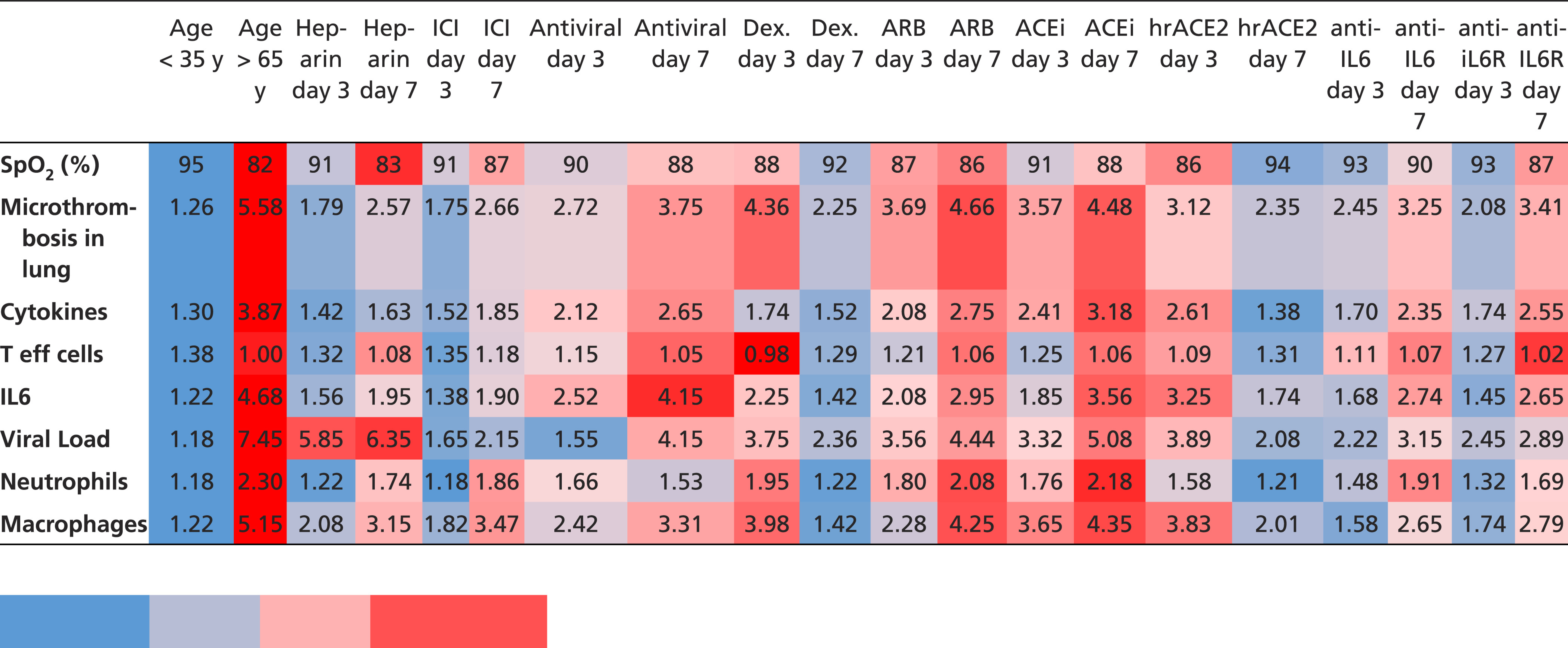

Favorable→unfavorable.

The table presents the results at the end of the simulation (day 20) for an old (>65 y old) male patient. Values have been normalized to the corresponding initial values, except for SpO_2_. Dex., dexamethasone.

Unsurprisingly, we found that heparin decreases thrombus formation and improves oxygenation when administered during early stages of disease but has no effect on viral load. ICI is also predicted to improve outcomes when initiated early. The antiviral therapy is effective in reducing viral load in early stages, but is less effective in reducing thrombus formation and improving oxygenation. Interestingly, dexamethasone is helpful when started later, but early administration can prevent the production of activated T cells, making it difficult to reduce the viral load. This mirrors the data from the Randomised Evaluation of COVID-19 Therapy trial, where dexamethasone was demonstrated to improve outcomes in patients requiring ventilatory support, but not in those with milder symptoms. These simulations also identify IL-6 directed therapies as potential therapeutics that may benefit patients if started early in the disease course. As far as inhibitors of the RAS are concerned, both ARBs and ACEis show modest benefits only when administered early. Furthermore, the model predicts that delayed administration of hrACE2 (on day 7 rather than day 3) will improve outcome. This is because the model assumes that the virus has already infected the lung at the start of the simulations, so hrACE2 has little influence on spread of virus within the lung; instead, its major activity in the simulations is to decrease cytokine production and thrombosis in the later stage of the disease.

### Optimizing Clinical Management of COVID-19 by Combining Therapies.

**Fig. 4.**
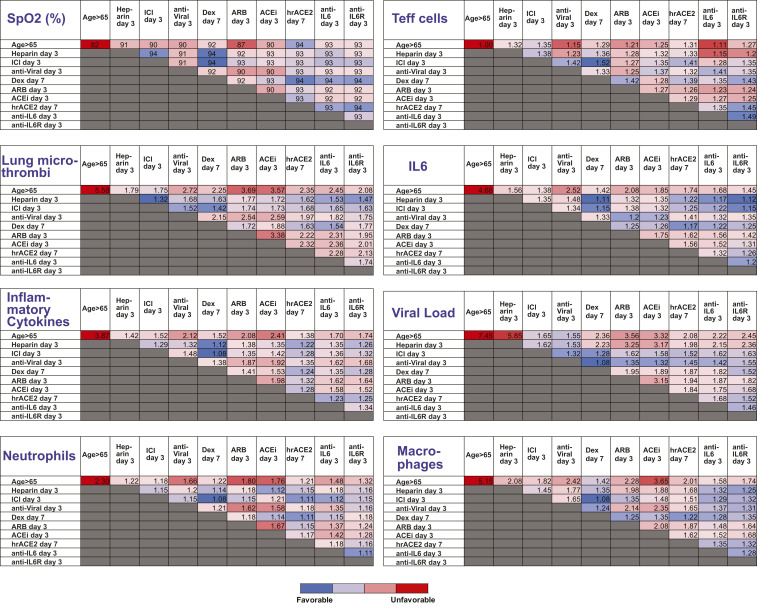
Summary of model predictions for the therapeutic outcome of combined treatments for male patients > 65 y old. The tables present results at the end of the simulation (day 20). Values have been normalized to the corresponding initial values except for SpO_2_. Dex., dexamethasone.

Because of the diverse mechanisms involved in COVID-19 pathogenesis, clinical management currently involves combination of multiple therapies in an effort to optimize therapeutic outcomes. To investigate the effects of combination treatments, we performed simulations combining various therapeutic approaches in pairs, using the best treatment time for each single treatment (i.e., day 3 or day 7; Table 1). We also accounted for older patients and those with comorbidities and dysregulated immune responses. Fig. 4 presents a summary of the combined treatments for older patients (>65 y); the results for the other COVID-19 phenotypes are presented in *SI Appendix*, Tables S3–S6. According to the model predictions, effective clinical management of older patients (with some baseline inflammation and impaired adaptive immunity) involves combination of heparin or ICI (day 3) with dexamethasone (day 7), which can improve oxygenation and decrease microthrombosis significantly; however, these treatments have little effect on viral load. For patients with obesity or hypertension, the combination of heparin with dexamethasone is again beneficial compared to other treatments. In addition, the use of anti-IL6/anti-IL6R therapy could also be considered in both of these patient populations. For diabetic patients, the combination of heparin or ICI with dexamethasone can improve oxygenation and thrombus formation similar to the other scenarios. Other treatments predicted to be beneficial for this phenotype are the combination of dexamethasone or ICI with hrACE2 or anti-IL6/anti-IL6R. These combination therapies in diabetic patients can treat hypoxemia and coagulation but also reduce the viral load. For patients with dysregulated immune systems, early blockade of IL6 combined with dexamethasone on day 7 gives optimal results; anti-IL6/anti-IL6R combined with antiviral therapy and/or heparin could also be considered.

## Discussion

COVID-19 is a complex disease that can affect multiple organs and is characterized by extremely heterogeneous presentation. As with other critical care syndromes such as septic shock and non-COVID-19 acute respiratory distress syndrome, disease severity results from a complex interplay of viral replication, adaptive and maladaptive immune response, and patient comorbidities. Such heterogeneity is a challenge to the development of effective therapies, as candidate approaches may elicit different responses depending on the phase of illness or patient comorbidities. Because of the complexity of the underlying mechanisms and interactions among the various components involved in the disease, the response of COVID-19 patients to any treatment is not intuitive, and, for that reason, we developed a highly sophisticated mathematical model to provide insights into the underlying mechanisms and predict optimal treatment strategies.

A main conclusion of our study is that disease progression and outcome of any treatment largely depends on the response rate of activated CD8^+^ T cells and subsequent control of the innate immune system response. A sustained activation of CD8^+^ T cells along with the control of the populations of neutrophils and macrophages is associated with a decrease in viral load and inflammation. This, in turn, will improve arterial oxygen saturation levels due to the decreased endothelial damage and microthrombosis, and limited formation of NETs (47). In line with these conclusions about the underlying mechanisms of the disease, our model predicts that antiviral and antiinflammatory drugs that were first employed to treat COVID-19 might have limited efficacy, depending on the stage of the disease progression. Furthermore, our simulations suggest that an optimal approach would be to enhance the adaptive immune response in the early stages while limiting harmful inflammation in the later stages of the disease. We also found that addition of heparin to the treatment regimen of COVID-19 can improve therapeutic outcomes. In case of patients with comorbidities (obesity, diabetes, hypertension) or dysregulated immunity, the treatment regimen could further include antiinflammatory drugs (e.g., anti-IL6/anti-IL6R) and RAS inhibitors and hrACE2 for the diabetic patients, in particular. Interestingly, the fact that progression of viral infections can be generalized as the result of interactions between CD8^+^ T cells and antigens has been shown by mathematical models focused on other types of viruses, such as hepatitis C, HIV-1, and simian HIV (48–51).

The activation of T cells also played a critical role in the progression of previous coronavirus infections, such as SARS-CoV and Middle East respiratory syndrome CoV, and the severity of these infections has been associated with type I interferon (52, 53). Moreover, the preventive effects of interferon are being tested for SARS-CoV-2 infection (Clinical Trial NCT04320238), although results have been mixed (25). Our model includes the generation of interferon by infected cells, and this interferon affects T cell activation as well as virus replication (*SI Appendix*, Eqs. **S34**, **S36**, **S55**, **S60**). To investigate the effect of interferon, we simulated interferon modulation treatment for the case of the older, at-risk male patient (>65 y). Our model predicts a protective effect of the treatment, which was slightly better when treatment was initiated at day 3 compared to day 7 (*SI Appendix*, Table S7).

Our mathematical modeling framework is subject to certain limitations. The model is able to account for the currently known components and interactions involved in disease pathogenesis but results in a large number of model parameters. Most of the values of these parameters were taken from the literature. However, because COVID-19 is a new disease, many of the parameter values are not known yet. For these parameters, it was necessary to make reasonable assumptions about their values, as summarized in *SI Appendix*, Table S1. We further validated the model predictions quantitatively with available clinical data for healthy and infected humans and qualitatively based on clinical observations for the different COVID-19 phenotypes. Our mathematical framework can be further refined as new mechanisms and more data become available and can be adapted for other diseases similar to COVID-19. Furthermore, our model is deterministic, and thus it does not incorporate uncertainties in the values of the model parameters, or the initial values employed. The main objective of this work was to provide a mathematical framework to characterize the multifaceted implications of COVID-19 on the human body. Uncertainty can be incorporated into the model by choosing the initial values of the model variables or the model parameters through a distribution; the distribution is sampled a number of times, and a Monte Carlo sampling method is employed for the solution of the equations. Also, the model only accounts for the temporal evolution of the infection and not for spatial effects in the lung or other organs, which will be considered in future studies.

Another limitation of the model is that it does not account for the humoral immune response, although circulating antibodies are important in viral control in COVID-19 (54). Future model development should include this additional complexity of antiviral immunity, as well as modulation of the immune system by vaccines. Also, the model does not account for adverse effects of the various treatments considered in this study, which could affect the therapeutic outcome. For instance, heparin use might be associated with hemorrhage, and RAS inhibitors can reduce blood pressure. Even though incorporation of adverse effects would be important, they would not affect the basic conclusions of the study and were omitted to avoid adding further complexity to the model. In conclusion, this study presents a mathematical representation of the known mechanisms of COVID-19 and could be utilized by the scientific community as a useful tool for further understanding the disease and investigating the benefits of treatments on a patient-specific basis.

## Materials and Methods

A detailed description of model equations, the description and values of model parameters, and the solution strategy are provided in *SI Appendix*. The mathematical model consists of two components: a detailed model of lung infection by the SARS-CoV-2 virus and a PK/PD model of COVID-19 infection and thrombosis to simulate events that happen throughout the body (Fig. 1). The lung model incorporates the infection of epithelial and endothelial cells by the SARS-CoV-2 virus through ACE2 modulation and activity, the release of proinflammatory cytokines, and the entire RAS. Cytokines that are central to COVID-19 (e.g., IL-6) are explicitly accounted for in the model, incorporating all known signaling pathways, such as the canonical and trans signaling pathway of IL-6 and its interaction with IL-6r and soluble IL-6r. Additional innate immune cells, specifically, neutrophils and macrophages, are incorporated into our model along with the interaction of immune cells with viral particles and infected cells as well as the formation of NETs. The model incorporates the recruitment of cytotoxic T cells by the infected cells and by IL-6 as well as the virus killing of cytotoxic T cells. In addition, the model accounts for the role of immune cells in modulating the expression of proinflammatory cytokines (55). Subsequent events, such as the impairment of the vascular network owing to the infection of endothelial cells by the SARS-CoV-2 virus and the resulting changes in blood oxygenation, are also included.

To study how the virus affects systemic events, such as inflammation and thrombosis, we coupled the lung model with a PK/PD model of viral infection. The PK/PD model represents the major organs of the body as compartments connected in an anatomical manner by the blood and lymphatic circulations (*SI Appendix*, Fig. S1). The organ compartments are then further subdivided into vascular and extravascular subcompartments, and each organ has a draining lymph node compartment. Briefly, the main transport processes for the biochemical species include 1) convective and diffusive transport across capillary walls, 2) reversible, nonsaturable, nonspecific binding in the extravascular compartments, and 3) reversible, and saturable, specific binding of virus to endothelial cells, and probabilistic infection of bound cells. Any infected cell generates microthrombi, which enter the circulation and can accumulate in target organs including brain, heart, and lung. Each species can be produced, bound, or degraded within each compartment. Blood flow is distributed according to known flows through each organ, and the overall mass balance allows calculation of the concentration of each species in each compartment over time. The PK/PD model includes the following key processes involved in the trafficking of viruses: 1) transport from the lung via the systemic circulation, 2) entry into endothelial cells via binding to ACE2, 3) entry into the cell and replication, and 4) exit from the cell and entry into the blood circulation. The important outputs of the model are the level of thrombosis in each organ and the viral load. When coupled with the lung model, we are able to analyze the dynamics of these readouts in light of local lung pathologies and predicted cytokine levels.

The model consists of a set of partial and ordinary differential equations. The values of the model parameters are summarized in *SI Appendix*, Table S1. We simulated COVID-19 infection and progression within a period of 20 d. The model does not account for the first stages of virus infection of the upper respiratory tract but from the time the virus has infected the lungs. Therefore, day 0 of the simulations corresponds to the initiation of lung infection. For the formulation of the model and the solution of the equations, the commercial finite elements software COMSOL Multiphysics v.5.5 was used. The computational finite element mesh employed in the present study consisted of 2,440 elements resulting in 1,201,044 degrees of freedom. The solution was tested and found to be mesh independent, the PARDISO solver was selected for the solution of the model equations, and the total solution time was ∼30 min.

### Code Availability.

The COMSOL code is available at Zenodo (56).

## Supplementary Material

Supplementary File

## Data Availability

All data supporting the findings of this study are available in the paper and *SI Appendix*.
