# Supplementary material for: In silico dynamics of COVID-19 phenotypes for optimizing clinical management
Source: Proc Natl Acad Sci U S A. 2021 Jan 5;118(3):e2021642118. doi: 10.1073/pnas.2021642118 (PMC7826337; doi:10.1073/pnas.2021642118)
Supplement: Supplementary File [file pnas.2021642118.sapp.pdf]

PNAS
www.pnas.org

Supplementary Information for
In silico dynamics of COVID-19 phenotypes for optimizing clinical management

Chrysovalantis Voutouri[a,1], Mohammad Reza Nikmaneshi[b,c,1], C. Corey Hardin[d], Ankit B. Patel[e], Ashish Verma[e], Melin J. Khandekar[f], Sayon Dutta[g], Triantafyllos Stylianopoulos[a,2], Lance L. Munn[c,2] and Rakesh K. Jain[c,2]

[1] C.V. and M.R.N. contributed equally

[2]To whom correspondence may be addressed: T.S. tstylian@ucy.ac.cy, L.L.M. munn@steele.mgh.harvard.edu, and R.K.J. jain@steele.mgh.harvard.edu

This PDF file includes:

- Supplementary text
- Figures S1 to S9 (not allowed for Brief Reports)
- Tables S1 to S7 (not allowed for Brief Reports)
- SI References

www.pnas.org/cgi/doi/10.1073/pnas.2021642118

SUPPLEMENTARY INFORMATION

Description of the Mathematical Model.

Pharmacokinetic-pharmacodynamic (PKPD) model

In the pharmacokinetic-pharmacodynamic (PKPD) model, each organ contains vascular and interstitial compartments (Supplementary Figure 1)(1-5). Blood flows through the vascular compartments and transport of fluid, solutes and particles occurs across the vessel walls into the extravascular tissue compartments. We assume that virus enters the systemic circulation from the lung and that virus binds to and infects endothelial cells in other organs. We do not consider infection of extra-pulmonary epithelium or direct spread through the GI tract. In non-pulmonary tissue, the virus can be i) flowing in the bloodstream, ii) bound to endothelial ACE2 receptors or iii) internalized in the endothelial cells. A cell infected with virus upregulates pathogen-associated and damage-associated molecular pattern pathways that result in upregulation of inflammatory cytokines. Damage to endothelium by virus and anti-viral immune cells also activates thrombosis and intrinsic and extrinsic coagulation pathways, creating intravascular microthrombi that can travel in the systemic circulation. The binding of virus to ACE2 also decreases ACE2 enzymatic activity.

Here, we first present the governing equations for each compartment of the multi-compartment model and then details about the microscale model of the lung.

In the PKPD model, Q_i is arterial blood flow rate, and L_i is lymphatic flow rate; thus, Q_i - L_i represents venous flow rate. The index i represents each organ/tissue -- lung, liver, spleen, G.I., kidney, torso, upper body, lower body, cardiac vessels, left ventricle of heart with arterial flow (*aHeart*), and right ventricle of heart with venous flow (*vHeart*). v_i is the free virus concentration, v_i^b the bound virus concentration, and v_i^{int} is the internalized virus concentration. T_i and T_i^b are the concentration of free and bound micro-thrombi, respectively, and V_i is the vascular volume of each compartment. a_i and a_i' are respectively the attachment rates of free virus to ACE2 and sACE2, d is detachment rate of bound virus from ACE2, mt_i, mt_i', mt_i'', and mt''' are maximum production rates of micro-thrombus in response to virus-ACE2 complex,IL6, cytokines, and neutrophil extracellular traps (NETs), respectively. dm_i is the attachment rate of micro-thrombus to vessels. ε_i is the dissolution rate of micro-thrombi due to thrombolysis, k_d is the inactivation rate of the virus, and p_{lung} is the proliferation rate of internalized virus. k_{acg}, k_{acg}', k_{acg}'' and k'''_{acg} are the micro-thrombus-inhibiting coefficients of anti-coagulation drugs for virus-ACE-2-induced coagulation, anti-coagulation drugs for IL6-induced coagulation, anti-coagulation drugs for cytokine storm-induced coagulation, and anti-coagulation drugs for NET-induced coagulation. k_{int} and k_a are the rates of bound virus internalization and virus release from the cell, respectively.

Heart compartment

In this model, the heart circulates blood through all the compartments. The heart compartment includes arterial and venous blood flows for both free viruses and micro-thrombosis. aHeart and vHeart refer to the left and right heart ventricles, respectively.

a) Free virus recirculation in arterial and venous blood flows into left and right heart ventricles, v_{aHeart} and v_{vHeart},

$$\frac{dv_{aHeart}}{dt}=\begin{pmatrix} (Q_{lung}-L_{lung})v_{lung} \\ -(Q_{liver}+L_{spleen}+L_{G.I.}+Q_{kidney} \\ +Q_{Torso}+Q_{lower\ body}+Q_{uppe\ body}+Q_{brain} \\ +Q_{cardic\ vessels})v_{aHeart} \end{pmatrix}/V_{aHeart} \quad (1a)$$

$$\frac{dv_{vHeart}}{dt}=\begin{pmatrix} (Q_{liver}-L_{liver})v_{liver}+(Q_{Torso}-L_{Torso})v_{Torso} \\ +(Q_{upper\ body}-L_{upper\ body})v_{upper\ body} \\ +(Q_{lower\ body}-L_{lower\ body})v_{lower\ body} \\ +(Q_{cardic\ vessels}-L_{cardic\ vessels})v_{cardic\ vessels} \\ +(Q_{brain}-L_{brain})v_{brain} \\ +(Q_{kidney}-L_{kidney})v_{kidney} \\ -Q_{lung}v_{vHeart} \end{pmatrix}/V_{vHeart} \quad (1b)$$

b) Arterial and venous recirculation of micro-thrombi, T_{aHeart} and T_{vHeart},

$$\frac{dT_{aHeart}}{dt}=\begin{pmatrix} (Q_{lung}-L_{lung})T_{lung} \\ -(Q_{liver}+L_{spleen}+L_{G.I.}+Q_{kidney} \\ +Q_{Torso}+Q_{lower\ body}+Q_{uppe\ body}+Q_{brain} \\ +Q_{cardic\ vessels})T_{aHeart} \end{pmatrix}/V_{aHeart} \quad (2a)$$

$$\frac{dT_{vHeart}}{dt} = \begin{pmatrix} (Q_{liver} - L_{liver})T_{liver} + (Q_{Torso} - L_{Torso})T_{Torso} \\ +(Q_{upper\ body} - L_{upper\ body})T_{upper\ body} \\ +(Q_{lower\ body} - L_{lower\ body})T_{lower\ body} \\ +(Q_{cardic\ vessels} - L_{cardic\ vessels})T_{cardic\ vessels} \\ +(Q_{brain} - L_{brain})T_{brain} \\ +(Q_{kidney} - L_{kidney})T_{kidney} \\ -Q_{lung}T_{vHeart} \end{pmatrix} / V_{vHeart} \tag{2b}$$

Liver

The liver compartment includes the hepatic portal vein from G.I. and spleen, and the hepatic artery to transport both free virus and micro-thrombi.

a) Free virus in liver vessels: hepatic portal vein from G.I. and spleen, and hepatic artery

$$\frac{dv_{liver}}{dt} = \begin{pmatrix} (Q_{G.I.} - L_{G.I.})v_{G.I.} + (Q_{spleen} - L_{spleen})v_{spleen} \\ +(Q_{liver} - Q_{G.I.} - Q_{spleen} + L_{G.I.} + L_{spleen})v_{aHeart} \\ -(Q_{liver} - L_{liver})v_{liver} \\ -a'_{liver}\ v_{liver}\left[sACE2\right]V_{liver} \\ -a_{liver}\ v_{liver}\left[ACE2\right]_{liver}\ V_{liver} \\ +d_{liver}\ v^{b}_{liver}\ V_{liver} \\ -k_{d}\ v_{liver}\ V_{liver} \\ +k_{a}\ v^{\text{int}}_{liver} V_{liver} \end{pmatrix} / V_{liver} \tag{3a}$$

b) Virus bound to liver vessel wall

$$\frac{dv^{b}_{liver}}{dt} = \begin{pmatrix} a_{liver}\ v_{liver}\left[ACE2\right]_{liver}\ V_{liver} \\ -d_{liver}\ v^{b}_{liver}\ V_{liver} \\ -k_{d}\ v^{b}_{liver}\ V_{liver} \\ -k_{\text{int}}\ v^{b}_{liver}\ V_{liver} \end{pmatrix} / V_{liver} \tag{3b}$$

c) Virus internalized into liver vessel endothelium

$$\frac{dv_{liver}^{\text{int}}}{dt}=\begin{pmatrix} p_{liver}\,k_{avp}\,v_{liver}^{b} \\ +k_{\text{int}}\,v_{liver}^{b}\,V_{liver} \\ -k_{a}\,v_{liver}^{\text{int}}V_{liver} \end{pmatrix}/V_{liver} \tag{3c}$$

d) Micro-thrombus formation and transport in liver

$$\frac{dT_{liver}}{dt}=\begin{pmatrix} (Q_{G.I.}-L_{G.I.})T_{G.I.}+(Q_{spleen}-L_{spleen})T_{spleen} \\ +(Q_{liver}-Q_{G.I.}-Q_{spleen}+L_{G.I.}+L_{spleen})T_{aHeart} \\ -\left(Q_{liver}-L_{liver}\right)T_{liver} \\ +mt_{liver}\,k_{acg}\,\dfrac{v_{liver}^{\text{int}}}{\left[ACE2\right]_{liver}+1}V_{liver} \\ +mt'_{liver}\,k'_{acg}\,\dfrac{[IL6]}{[IL6]+1}V_{liver} \\ +mt''_{liver}\,k''_{acg}\,\dfrac{[c]}{[c]+1}V_{liver} \\ -\varepsilon_{liver}\,T_{liver}\,V_{liver}-dm_{liver}\,T_{liver}\,V_{liver} \end{pmatrix}/V_{liver} \tag{3d}$$

e) Micro-thrombus accumulation in liver

$$\frac{dT_{liver}^{b}}{dt}=\left(dm_{liver}\,T_{liver}\,V_{liver}-\varepsilon_{liver}\,T_{liver}^{b}\,V_{liver}\right)/V_{liver} \tag{3e}$$

f) ACE2 density of liver

$$\frac{d\left[ACE2\right]_{liver}}{dt}=d_{liver}\,v_{liver}^{b}-a_{liver}\,v_{liver}\left[ACE2\right]_{liver} \tag{3f}$$

For the ith organ/tissue, where i = spleen, G.I., upper body, lower body, torso and cardiac vessels

All these compartments have upstream blood flow coming from the left ventricle of heart and downstream blood flow going back to the right ventricle of heart to allow both free viruses and micro-thrombosis to circulate in the whole body.

a) Free virus in the vessels of ith organ

$$\frac{dv_i}{dt} = \begin{pmatrix} Q_i\, v_{aHeart} - (Q_i - L_i)\, v_i \\ -a_i'\, v_i \left[sACE2 \right] V_i \\ -a_i\, v_i \left[ACE2 \right]_i\, V_i \\ +d_i\, v_i^b\, V_i \\ -k_d\, v_i\, V_i \\ +k_a\, v_i^{\mathrm{int}}\, V_i \end{pmatrix} / V_i \tag{4a}$$

b) Bound virus on vessel wall of ith organ

$$\frac{dv_i^b}{dt} = \begin{pmatrix} a_i\, v_i \left[ACE2 \right]_i\, V_i \\ -d_i\, v_i^b\, V_i \\ -k_d\, v_i^b\, V_i \\ -k_{\mathrm{int}}\, v_i^b\, V_i \end{pmatrix} / V_i \tag{4b}$$

c) Internalized virus in endothelium of ith organ

$$\frac{dv_i^{\mathrm{int}}}{dt} = \begin{pmatrix} p_i\, k_{avp}\, v_i^b \\ +k_{\mathrm{int}}\, v_i^b\, V_i \\ -k_a\, v_i^{\mathrm{int}}\, V_i \end{pmatrix} / V_i \tag{4c}$$

d) Micro-thrombus formation and transport in i[th] organ

$$\frac{dT_i}{dt} = \begin{pmatrix} Q_i\, T_{aHeart} - \left(Q_i - L_i \right) T_i \\ +mt_i\, k_{acg}\, \dfrac{v_i^{\mathrm{int}}}{\left[ACE2 \right]_i + 1} V_i \\ +mt_i'\, k_{acg}'\, \dfrac{[IL6]}{[IL6]+1} V_i \\ +mt_i''\, k_{acg}''\, \dfrac{[c]}{[c]+1} V_i \\ -\varepsilon_i\, T_i\, V_i \end{pmatrix} / V_i \tag{4d}$$

e) Micro-thrombus accumulation in i[th] organ

$$\frac{dT_i^b}{dt} = \left(dm_i\, T_i\, V_i - \varepsilon_i\, T_i^b\, V_i \right) / V_i \tag{4e}$$

f) ACE2 density of ith organ

$$\frac{d[ACE2]_i}{dt} = d_i\, v_i^b - a_i\, v_i\,[ACE2]_i \tag{4f}$$

Lung

The lung compartment has upstream blood flow coming from heart and downstream blood flow going back to heart to transport both free viruses and micro-thrombi. Free, bound and internalized virus concentrations in the lung are calculated using the microscale model of the lung, Eqs. 33-35. ACE2 concentration of the lung is calculated by Eq. 32 of the microscale model of lung. The microthrombus dynamics are:

a) Micro-thrombus formation and transport in lung

$$\frac{dT_{\text{lung}}}{dt} = \begin{pmatrix} Q_{\text{lung}}\,T_{vHeart} - \left(Q_{\text{lung}} - L_{\text{lung}}\right)T_{\text{lung}} \\ +mt_{\text{lung}}\,k_{acg}\,\dfrac{v_{\text{lung}}^{\text{int}}}{[ACE2]_{\text{lung}}+1}V_{\text{lung}} \\ +mt'_{\text{lung}}\,k'_{acg}\,\dfrac{[IL6]}{[IL6]+1}V_{\text{lung}} \\ +mt''_{\text{lung}}\,k''_{acg}\,\dfrac{[c]}{[c]+1}V_{\text{lung}} \\ +mt'''\,k'''_{acg}\,\dfrac{[\text{NETs}]}{[NETs]+1}V_{\text{lung}} \\ -\varepsilon_{\text{lung}}\,T_{\text{lung}}\,V_{\text{lung}} \end{pmatrix} / V_{\text{lung}} \tag{5a}$$

b) Micro-thrombus accumulation in lung

$$\frac{dT_{\text{lung}}^b}{dt} = \left(dm_{\text{lung}}\,T_{\text{lung}}\,V_{\text{lung}} - \varepsilon_{\text{lung}}\,T_{\text{lung}}^b\,V_{\text{lung}}\right)/V_{\text{lung}} \tag{5b}$$

Mass conservation

$$\begin{aligned} \mathrm{Q}_{\text{lung}} = {} & \mathrm{L}_{\text{lung}} + \mathrm{Q}_{liver} + \mathrm{L}_{G.I.} + \mathrm{L}_{\text{spleen}} + \mathrm{Q}_{\text{kidney}} \\ & + \mathrm{Q}_{\text{Torso}} + \mathrm{Q}_{lower\,body} + \mathrm{Q}_{upper\ body} + \mathrm{Q}_{brain} + \mathrm{Q}_{cardiac\ vessels} \end{aligned} \tag{6}$$

Microscale Lung Model

Equations describing the Renin-Angiotensin system

The reaction of angiotensinogen (AGT) is governed by Eq. 7,(6)

$$\frac{d[AGT]}{dt} = K_{AGT} - c_{Renin}[AGT] - \frac{\ln 2}{h_{AGT}}[AGT] \tag{7}$$

The rate of change of angiotensinogen depends on its production (K_{AGT}); production of ANG I catalyzed by renin is assumed to follow first order kinetics, and thus PRA = c_{Renin}[AGT], degradation of AGT is considered to exhibit first-order kinetics in terms of its half-life h_{AGT}.

The mass balance for Renin is(6)

$$\frac{d[Renin]}{dt} = s_{Renin} + K_f([ANGII]_0 - [ANGII])\left(1 - \frac{[ANGII]_0 - [ANGII]}{f}\right) - \frac{\ln 2}{h_{Renin}}[Renin] \quad (8)$$

The first term, s_{Renin} , accounts for a constant source of renin from the kidney; the second term is the influence of ANG II negative feedback on renin production, [ANG II]$_0$ is the initial concentration of ANG II, k_f and f are parameters for the feedback, and h_{Renin} is the half-life for the degradation of renin.

$$s_{Renin} = \frac{\ln 2}{h_{Renin}}[Renin]_0 \quad (9)$$

The renin source term is computed at steady state, where [Renin]$_0$ is the initial concentration of renin.

The mass balance for ANG I is(6)

$$\frac{d[ANGI]}{dt} = c_{Renin}[AGT] + K_{Renin}([Renin] - [Renin]_0) - K_{ACE}[ANGI] - K_{NEP}ANGI - K^{on}_{ACE2-ANGI}[ANGI][ACE2] + K^{off}_{ACE2-ANGI}[ACE2 - ANGI] - \frac{\ln 2}{h_{ANGI}}[ANGI] \quad (10)$$

where the first term represents the glucose-dependent renin-catalyzed contribution to the production of ANG I from AGT, the second term represents the change to ANG I synthesis from AGT due to the feedback of ANG II on renin with rate constant K_{Renin}, the third term is the ACE-catalyzed conversion of ANG I to ANG II and has a glucose-dependent rate parameter K_{ACE}, the fourth term is the consumption of ANG I to form ANG-(1-7) with the glucose-independent rate parameters K_{NEP}, the following two terms describe the binding/unbinding of ANG I on the ACE 2 receptors, and $h_{ANG\ I}$ is the half-life for the degradation of ANG I.

The mass balance for the free ANG II is(6)

$$\frac{d[ANGII]}{dt} = K_{ACE}[ANGI] - K^{on}_{AT1}[ANGII][AT1R] + K^{off}_{AT1}[AT1R - ANGII] - K^{on}_{AT2}[ANGII][AT2R] + K^{off}_{AT2}[AT2R - ANGII] - K_{APA}[ANGII] - K^{on}_{ACE2-ANGII}[ANGII][ACE2] + K^{off}_{ACE2-ANGII}[ACE2 - ANGII] - \frac{\ln 2}{h_{ANGII}}[ANGII] \quad (11)$$

where the first term is the production of ANG II in the presence of ACE, the following two terms

The mass balance for ANGII bound to AT2 receptor is(7)

$$\frac{d[AT2R-ANGII]}{dt}=K_{AT2}^{on}[ANGII][AT2R]-K_{AT2}^{off}[AT2R-ANGII]-\frac{\ln 2}{h_{AT2R-ANGII}}[AT2R-ANGII] \quad (13)$$

where the first two terms describe the binding/unbinding of ANG II on the AT2 receptor and has a degradation half-life $h_{AT2R\text{-}ANGII}$.

The mass balance for ANG(1-7) is(7)

$$\frac{d[ANG_{(1-7)}]}{dt}=K_{NEP}\,[ANGI]+K_{ACE2}\,[ACE2-ANGII]+K_{ANG(1-9)}\,[ANG(1-9)]-K_{MAs}^{on}[ANG(1-7)][MAsR] + K_{MAs}^{off}[MAsR-ANG(1-7)]-\frac{\ln 2}{h_{ANG_{(1-7)}}}[ANG_{(1-7)}] \quad (14)$$

where the first three terms are the production of ANG(1-7) by ANGI, ANGII bound to ACE2, and by ANG(1-9), the following two terms describe the binding/unbinding of ANG 1-7 to the MAs receptors and $h_{ANG\ 1\text{-}7}$ is the half-life for degradation of ANG 1-7.

The mass balance for ANG(1-7) bound to Mas receptor is

$$\frac{d[MAsR-ANG(1-7)]}{dt} = K_{MAs}^{on}[ANG(1-7)][MAsR]-K_{MAs}^{off}[MAsR-ANG(1-7)]-\frac{\ln 2}{h_{MAsR-ANG(1-7)}}[MAsR - ANG(1-7)] \quad (15)$$

where the first two terms describe the binding/unbinding of ANG 1-7 on the MAs receptor and has a degradation half-life of $h_{MasR\text{-}ANG1\text{-}7}$.

The mass balance of ANG (1-9) is(7)

$$\frac{d[ANG(1-9)]}{dt}=K_{ACE2}[ACE2-ANGI]-\frac{\ln 2}{h_{ANG(1-9)}}[ANG(1-9)] \quad (16)$$

where the first term is the production of ANG(1-9) by ANGI bound to the ACE2 and $h_{ANG\ 1\text{-}9}$ is the half-life for degradation of ANG 1-9.

The mass balance term of ANGIII is(7)

$$\frac{d[ANGIII]}{dt}=K_{APA}\,[ANGII]\ -K_{APM}\,[ANGIII]-\frac{\ln 2}{h_{ANGIII}}[ANGIII] \quad (17)$$

where the first term is the production of ANGIII by ANG II, the second term describes the production of ANGIV by ANGIII and $h_{ANG\ III}$ is the half-life for degradation of ANG III.

The mass balance for ANGIV is(8)

$$\frac{d[ANGIV]}{dt} = K_{APM}\,[ANGIII]\; - K_{AT4}^{on}[ANGIV][AT4R] + K_{AT4}^{off}[AT4R - ANGIV] + -\frac{\ln 2}{h_{ANGIV}}[ANGIV] \qquad (18)$$

where the first term is the production of ANG IV by ANG III, the following two terms describe the binding/unbinding of ANG IV on the AT4 receptor and h_{ANGIV} the half-life for degradation of ANG IV.

The mass balance for ANGIV bound to AT4 receptor is

$$\frac{d[AT4R - ANGIV]}{dt} = K_{AT4}^{on}[ANGIV][AT4R] - K_{AT4}^{off}[AT4R - ANGIV] - \frac{\ln 2}{h_{AT4R-ANGIV}}[AT4R - ANGIV] \qquad (19)$$

where the first two terms describe the binding/unbinding of ANG IV on the AT4 receptors and has a degradation half-life $h_{AT4R\text{-}ANGIV}$.

The mass balance for ANGI bound to ACE2 is

$$\frac{d[ACE2 - ANGI]}{dt} = K_{ACE2-ANGI}^{on}[ANGI][ACE2] - K_{ACE2-ANGI}^{off}[ACE2 - ANGI] - K_{ACE2}[ACE2 - ANGI] \qquad (20)$$

where the first two terms describe the binding/unbinding of ANGI on the ACE2 and the last term describes the degradation of ANGI bound to ACE2.

Mass balance for ANGII bound to ACE2:

$$\frac{d[ACE2-ANGII]}{dt} = K_{ACE2-ANGII}^{on}[ANGII][ACE2] - \; K_{ACE2-ANGII}^{off}[ACE2 - ANGII] - K_{ACE2}\,[ACE2 - ANGII] \qquad (21)$$

where the first two terms describe the binding/unbinding of ANGII on the ACE2 and the last term describes the degradation of the ANGII bound to ACE2.

The mass balance for AT1 receptor is

$$\frac{d[AT1R]}{dt} = S_{AT1R} - K_{AT1}^{on}[ANGII][AT1R] + K_{AT1}^{off}[AT1R - ANGII] - d_{AT1R}[AT1R] \qquad (22)$$

where the first term represents the source term for AT1 receptor, the following two terms describe the binding/unbinding of ANG II on the AT1 receptor, and the last term describes the degradation of the AT1 receptor.

The mass balance for AT2 receptor is

$$\frac{d[AT2R]}{dt} = S_{AT2R} - K_{AT2}^{on}[ANGII][AT2R] + K_{AT2}^{off}[AT2R - ANGII] - d_{AT2R}[AT2R] \qquad (23)$$

where the first term represents the source term for AT2 receptor, the following two terms describe the binding/unbinding of ANG II on the AT2 receptors, and the last term describes the degradation of the AT2 receptor.

The mass balance for MAs receptor is

$$\frac{d[MAsR]}{dt} = S_{MAsR} - K_{MAs}^{on}[ANG(1-7)][MAsR] + K_{MAs}^{off}[MAsR - ANG(1-7)] - d_{MAsR}[MAsR] \tag{24}$$

where the first term represents the source term for MAs receptor, the following two terms describe the binding/unbinding of ANG 1-7 on the MAs receptors, and the last term describes the degradation of the MAs receptor.

The mass balance for AT4 receptor is

$$\frac{d[AT4R]}{dt} = S_{AT4R} - K_{AT4}^{on}[ANGIV][AT4R] + K_{AT4}^{off}[AT4R - ANGIV] - d_{AT4R}[AT4R] \tag{25}$$

where the first term represents the source term for AT4 receptor, the following two terms describe the binding/unbinding of ANG IV on the AT4 receptors, and the last term describes the degradation of the AT4 receptor.

Mass Balance Equations for IL6, IL6 receptor, soluble IL6R, binding of IL6 to IL6R and sIL6R and production of VEGF

The mass balance for IL-6 is

$$\frac{d[IL6]}{dt} = K_{IL6}M_a\mathrm{V} - \gamma_{IL6}[IL6] - K_{IL6}^{on}[IL6][IL6R] + K_{IL6}^{off}[IL6R - IL6] + K_{sIL6R}^{off}[sIL6R - IL6] \tag{26}$$
$$-K_{sIL6R}^{on}[sIL6R][IL6] + K_{IL6-Tn}T_N + K_{IL6-IN}In + K_{IL6-IEC}[IEC] + K_{IL6-TE}T_E$$

where the first term is the production of IL-6 by virus-activated macrophages, the second term is the degradation of the IL-6, the following two terms describe the binding/unbinding of IL-6 to the IL-6 receptor, the following two terms describe the binding/unbinding of IL-6 to the soluble IL-6 receptor, and the following four terms describe the production by Naïve T cells, infected epithelial cells, infected endothelial cells and Effector (Activated) T cells.

The mass balance for IL-6 receptor

$$\frac{d[IL6R]}{dt} = S_{IL6R} - K_{IL6}^{on}[IL6][IL6R] + K_{IL6}^{off}[IL6R - IL6] - K_{sIL6R}[IL6R] - d_{IL6R}[IL6R] \tag{27}$$

where the first term represents the source term for IL-6 receptor, the following two terms describe the binding/unbinding of IL-6 on the IL-6 receptors and the last term is the degradation rate.

The mass balance for soluble IL-6 receptor

$$\frac{d[sIL6R]}{dt} = K_{sIL6R}[IL6R] - K_{sIL6R}^{off}[sIL6R - IL6] \tag{28}$$

where the first term is the production of soluble IL-6 by IL-6 receptor and the last describe the unbinding of IL6 from the soluble IL-6 receptor.

The mass balance for IL-6 bound to IL-6 receptor is

$$\frac{d[IL6R - IL6]}{dt} = K_{IL6}^{on}[IL6][IL6R] - K_{IL6}^{off}[IL6R - IL6] - \frac{\ln 2}{h_{IL6R}}[IL6R - IL6] \tag{29}$$

where the first two terms describe the binding/unbinding of IL-6 on the IL-6 receptor and has a degradation half-life h_{IL6R}

The mass balance for IL-6 bound to soluble IL-6 receptor

$$\frac{d[sIL6R - IL6]}{dt} = K_{sIL6R}^{on}[sIL6R][IL6] - K_{sIL6R}^{off}[sIL6R - IL6] \tag{30}$$

where the first two terms describe the binding/unbinding of IL-6 to soluble IL-6 receptor.

The mass balance for VEGF

$$\frac{d[VEGF]}{dt} = S_{VEGF} + K_{VEGF}[sIL6R - IL6] - d_{VEGF}[VEGF] + \gamma_{VEGF}(100 - SPO2) \tag{31}$$

where the first term represents the source term of VEGF, the second term describes the production of VEGF by IL-6 bound on the soluble IL-6 receptor, the third term is the degradation rate of the VEGF and the last term describes the production of VEGF by hypoxia

Model equations for virus infection and immune cells activation

The mass balance for soluble ACE2 receptor

$$\frac{d[sACE2]}{dt} = -K_{sACE2}[sACE2][V] - \sum_i K_{sACE2}[sACE2][v_i] + K_{Adam17}[ACE2] - d_{sACE2}[sACE2] \tag{32}$$

where the first term is the binding of soluble ACE2 receptor to the virus, the third term is internalization everywhere, and the following term is the production of soluble ACE2 receptor by ACE2 receptor interaction through Adam17 and the last term is the degradation rate of the soluble ACE2 receptor.

The mass balance of ACE2 receptor is

$$\begin{aligned}\frac{d[ACE2]}{dt} &= S_{ACE2}([EC] + H) - K_{ACE2-Virus}^{on}[v][ACE2] + K_{ACE2-Virus}^{off}[v_b] - K_{Adam17}[ACE2] - \\ &K_{ACE2-ANGI}^{on}[ANGI][ACE2] + K_{ACE2-ANGI}^{off}[ACE2 - ANGI] - \\ &K_{ACE2-ANGII}^{on}[ANGII][ACE2] + K_{ACE2-ANGII}^{off}[ACE2 - ANGII]\end{aligned} \tag{33}$$

where the first term describes the production of ACE2 receptor by endothelial and epithelial cells, the second and third term describes the interaction with the virus, the fourth term describes the

production of soluble ACE2 and the rest terms describe the interaction of ACE2 with ANGI and ANGII.

The virus can be in three states, the free virus that diffuses in the lung tissue, the bound virus on the epithelial cells of the lungs and the internalized virus. The equations for the three states of the virus are:

<u>Free virus</u>

$$\frac{dv}{dt} = Q_{lung}v/V_{lung} - (Q_{lung} - L_{lung})v/V_{lung} - K_{sACE2}[v][sACE2] - K^{on}_{ACE2-Virus}[v][ACE2] + K^{off}_{ACE2-Virus}[v_b] - K_d v + K_a\left(1 - K_{IF}\ \frac{IF}{K_{IF} + IF}\right) v_{int} \tag{34}$$

Where the terms respectively describe convectional transport of free virus in lung, the binding of the virus to soluble ACE2, the binding of the virus to ACE2, the detachment of bound virus from ACE2, the inactivating of the virus, and the source of new viral particles from the release of the infected cells following cell death.

<u>Bound virus</u>

$$\frac{dv_b}{dt} = K^{on}_{ACE2-Virus}[v][ACE2] - K^{off}_{ACE2-Virus}[v_b] - K_d v_b - K_{int} v_b \tag{35}$$

where the first two terms describe the binding/unbinding of the virus on the ACE2 receptor, the third term is the degradation of the bound virus, the last term is the internalization of the bound virus into the cells.

<u>Internalized virus</u>

$$\frac{dv_{int}}{dt} = P_{lung}\ k_{avp}\ v_{int} + K_{int}v_b - K_a\ \left(1 - K_{IF}\ \frac{IF}{K_{IF} + IF}\right) v_{int} \tag{36}$$

where the first term describes replication, the second the internalization of the bound virus and the third term describes the viral particles that exit the cell.

<u>Pro-inflammatory cytokines:</u>

$$\frac{dc}{dt} = S_c v_{int} + K_{\mathrm{AT1}R}[\mathrm{AT1R} - \mathrm{ANGII}] + S_{\mathrm{n}}(N + M_a + In) - d_{\mathrm{s}}c - K_{AT1R-MAsR}[MAsR] - K_{AT1R-AT2R}[AT2R] + K_{c-IL6}[IL6 - sIL6R](K_{c-EC}[EC] + K_{c-H}H) \tag{37}$$

The cytokines are produced by the internalized virus, the ANGII which is bound to AT1R, as well as by the neutrophils (N), the macrophages (M_a) and the Infected cell (In), they are degraded by the Mas receptor and AT2 receptor, and they are produced by IL6 bound to IL6 receptor as well as the healthy endothelial and epithelial cells.

<u>Anti-inflammatory cytokines</u>

$$\frac{da}{dt} = K_g \varphi_a MN + K_{Ang1-7}[MAsR - ANG_{(1-7)}] - \gamma_a a \quad (38)$$

the first two term describe the production of anti-inflammatory cytokines by Neutrophils and ANG(1-7) bound to MasR and have a degradation rate of γ_a

<u>Infected epithelial cells(9)</u>

$$\frac{dIn}{dt} = K_b H v_{int} + \varphi_{\mathrm{c}} HN(K_H(c-a)+1) - \varphi_{CTL} In T_{\mathrm{E}} \quad (39)$$

Infected cells are produced by the virus entry into healthy cells and the neutrophil's killing of healthy cells and are killed by Cytotoxic T cells.

<u>Healthy epithelial cells(9)</u>

$$\frac{dH}{dt} = R_H H - K_b H v_{int} - \varphi_{\mathrm{c}} HN(K_H(c-a)+1) \quad (40)$$

where the first term describes the proliferation of healthy cells, the second term describes the production of infected cells by the virus entry into healthy cells and the last term describes the neutrophil's killing of healthy cells.

<u>Neutrophils(10)</u>

$$\frac{dN}{dt} = \frac{\chi_{\mathrm{n}}\mathrm{c}}{1+a} + \chi_{N-IL6}[IL6R - IL6] - \gamma_{\mathrm{n}} N * (1 + \beta_n \frac{v}{v+1}) \quad (41)$$

Neutrophils are recruited by cytokines and IL-6 which is bound to IL-6R, and the last term describes the production of Neutrophil extracellular traps

<u>Neutrophils Extracellular Traps (NETs)</u>

$$\frac{d[NETs]}{dt} = \gamma_{\mathrm{n}} N * \left(1 + \beta_n \frac{v}{v+1}\right) - \gamma_{NETs}[NETs] \quad (42)$$

where the first term describes the production Neutrophil extracellular traps and the last term is their degradation rate.

<u>Macrophages(10)</u>

$$\frac{dM_a}{dt} = \chi_{\mathrm{m}}\mathrm{c} + \chi_{Ma-IL6}[IL6R - IL6] - \gamma_{\mathrm{a}} M_a \quad (43)$$

Macrophages are recruited by cytokines and IL-6 which is bound to IL-6R, die with a rate constant γ_a

<u>Healthy Endothelial cells</u>

$$\frac{d[EC]}{dt} = K_{ec}[EC] - K_{ec}^{b}[EC] v_{int} \quad (44)$$

Where the first term describes the overall ("effective") proliferation/death of endothelial cells and the last term is the conversion of healthy endothelial cells to infected endothelial cells by internalized virus.

Infected Endothelial cells

$$\frac{d[IEC]}{dt} = K_{ec}^{b}[EC]v_{int} \quad (45)$$

Infected endothelial cells are produced by the virus entry into of healthy endothelial cells

Vascular density

$$S_v = \frac{[EC]}{[EC_o]}S_v^0 \quad (46)$$

where S_v^0 is the vascular density of the normal lung and $[EC_o]$ is the initial endothelial cell population.

Oxygen consumption rate(11)

$$V_{O2} = D_{LO2}(P_A - P_b) \quad (47)$$

where D_{LO2} is the lung diffusing capacity, P_A is the partial pressure of oxygen (P_{O2}) in alveolar air and P_b is the mean P_{O2} in pulmonary capillary.

The oxygen diffusion can be represented as two components in series, one associated with the alveolar membrane D_{MO2} and one associated with erythrocytes D_{eO2}

$$(D_{LO2})^{-1} = (D_{MO2})^{-1} + (D_{eO2})^{-1} \quad (48)$$

The membrane component is estimated as

$$D_{MO2} = K_{O2}\frac{\left(\frac{1}{2}\right)[S(A) + S(c)]}{\tau_{hb}} \quad (49)$$

where K_{O2} is the Krogh diffusion constant, $S(A)$ is the average of the alveolar surface area, $S(c)$ is the capillary surface area and τ_{hb} is the harmonic mean of the distance between the alveolar surface and the erythrocyte surface.

$$K_{O2} = \varphi_{KO2}\frac{[EC]}{[EC_o]}K_{02}^{0} \quad (50)$$

The erythrocyte component is calculated from

$$D_{eO2} = \theta_{O2}V(c) \quad (51)$$

where $V(c)$ is the pulmonary capillary blood volume and θ_{O2} is the oxygen unloading conductance of blood.

The partial pressure of oxygen (P_{O2}) in pulmonary capillary calculated from

$$P_b = P_A - \frac{V_{o2}^{max}}{D_{LO2}} \quad (52)$$

The oxyhemoglobin saturation according to the Hill equation

$$S(P_b) = \frac{\left(\frac{P_b}{P_{50}}\right)^n}{1 + \left(\frac{P_b}{P_{50}}\right)^n} \quad (53)$$

Naïve T cells(12)

$$\frac{dT_\mathrm{N}}{dt} = S_{TN} - h_{TE}T_N\left(\frac{As}{1+As}\right)\left(\frac{c}{1+c}\right)\left(\frac{IF}{K_{IF}+IF}\right)\frac{K_T}{K_T + [PD1 - PDL1]} - d_{TN}T_N \quad (54)$$

where the first term represents the source term of Naïve T cells, the second term is the conversion of Naïve T cells to Effector (Activated) T cells and the last term is the degradation of the Naïve T cells.

Effector (Activated) T cells(12, 13)

$$\frac{dT_\mathrm{E}}{dt} = h_{TE}T_N\left(\frac{As}{1+As}\right)\left(\frac{c}{1+c}\right)\left(\frac{IF}{K_{IF}+IF}\right)\frac{K_T}{K_T + [PD1 - PDL1]} - \varepsilon T_E\frac{[PD1 - PDL1]}{AS} \quad (55)$$

where the first is the conversion of Naïve T cells to Effector (Activated) T cells and the last term is the degradation of the Effector (Activated) T cells by the PD-1 bound to PD1 ligand.

PDL-1(12)

$$\frac{d[PDL1]}{dt} = [(\lambda_H - d_H)H + (\lambda_{EC} - d_{EC})[EC] + (\lambda_{In} - d_{In})In]\frac{[PDL1]}{H + [EC] + In} + h_{PDL1}T_E \quad (56)$$

where the first term describes the production(λ_i)/degradation(d_i) of the PD1 ligand by healthy epithelial cells, healthy endothelial cells and infected epithelial cells, the last term describes the production of the PD1 ligand by Effector (Activated) T cells

PD-1(12)

$$\frac{d[PD1]}{dt} = [(\lambda_{TE} - d_{TE})T_E + (\lambda_{TN} - d_{TN})T_N + (\lambda_N - d_N)N + (\lambda_{Ma} - d_{Ma})M_a]\frac{[PD1]}{T_E + T_N + N + M_a} - \mu_{PD1}[anti - PD1] \quad (57)$$

where the first describes the production(λ_i)/degradation(d_i) of the PD1 by Effector (Activated) T cells, Naïve T cells, neutrophils and macrophages, the last term describes the degradation of the PD1 by anti-PD1

PD-1 binding to PDL-1(12)

$$\frac{d[PD1-PDL1]}{dt}=a_{PL}[PD1][PDL1]-d_Q[PD1-PDL1] \tag{58}$$

where the first term describes the binding of PD1 to the PD1 ligand and the last term describes its degradation rate.

anti-PD-1

$$\frac{d[anti-PD1]}{dt}=\gamma_A-\mu_A[PD1][anti-PD1]-d_A[anti-PD1] \tag{59}$$

where the first term represents the source term of the anti-PD1, the second term describes the degradation of the anti-PD1 by PD1 and the last term describes its degradation rate.

Interferon (IF)

$$\frac{dIF}{dt}=a_{IF}\,(IEC+In)\;-\;\varepsilon_{IF}\,IF \tag{60}$$

where the first term describes the production of interferon by infected endothelial and epithelial cells and the last term describes the degradation rate of interferon.

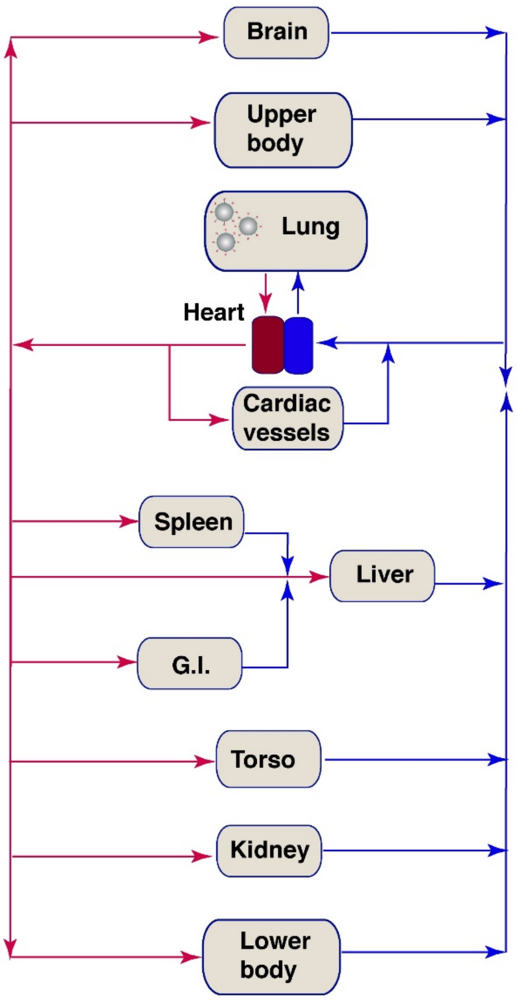

Fig. S1. PK/PD model of COVID-19 infection and thrombosis simulates events that occur throughout the body when a patient contracts COVID-19. Virus exits the lung via the systemic circulation and can infect endothelial cells (ECs) in various organs, modeled here as well-mixed compartments. Upon infection, viral replication and cell death cause vessel damage and local inflammation that induce thrombosis. Each tissue can then become a source of micro-thrombi, which enter the systemic circulation and can accumulate in the microvessels of the heart, lung and brain, inducing ischemic events.

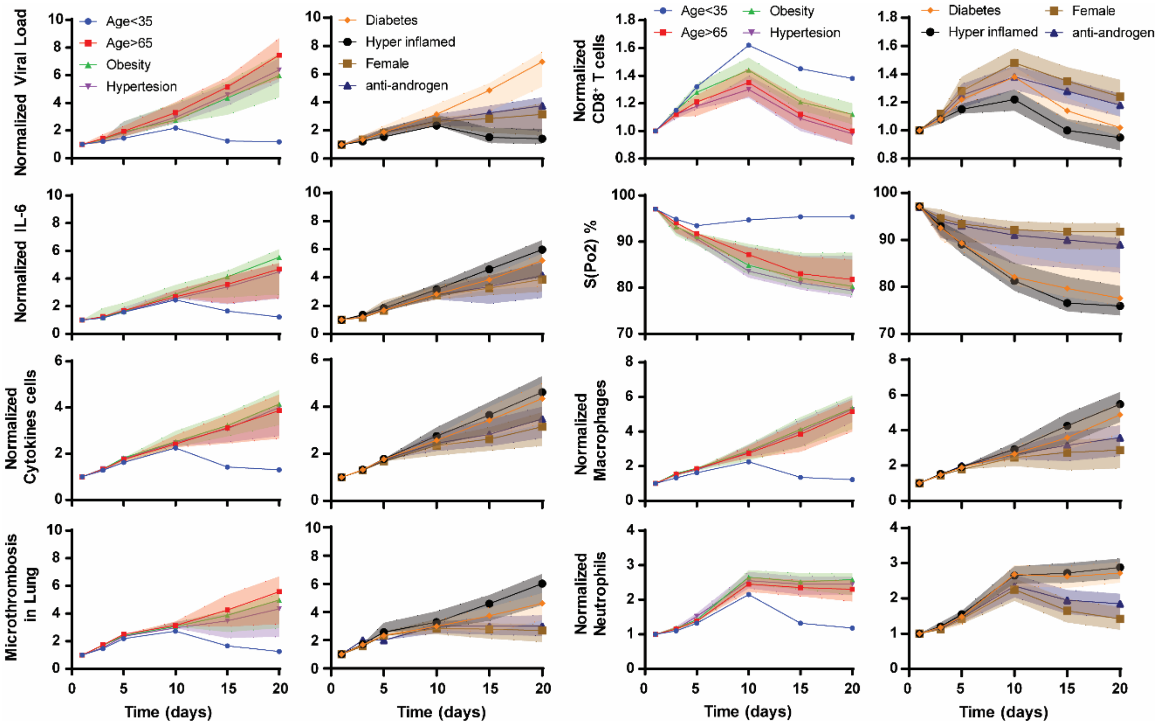

Fig. S2. Sensitivity analyses an older male patient (>65 years old), an older female patient (>65 years old) and older male patients with co-morbidities: hypertension, obesity and diabetes, as well as for hyper-inflamed patients. For the analysis, we varied simultaneously the values of all parameters involved in the various patient phenotypes (Supplemental Table S2) by a factor of 10, 100 and 1000. The plots depict the range of values of the model variables. The baseline simulation for the young male patient (<35 years old) is shown for comparison.

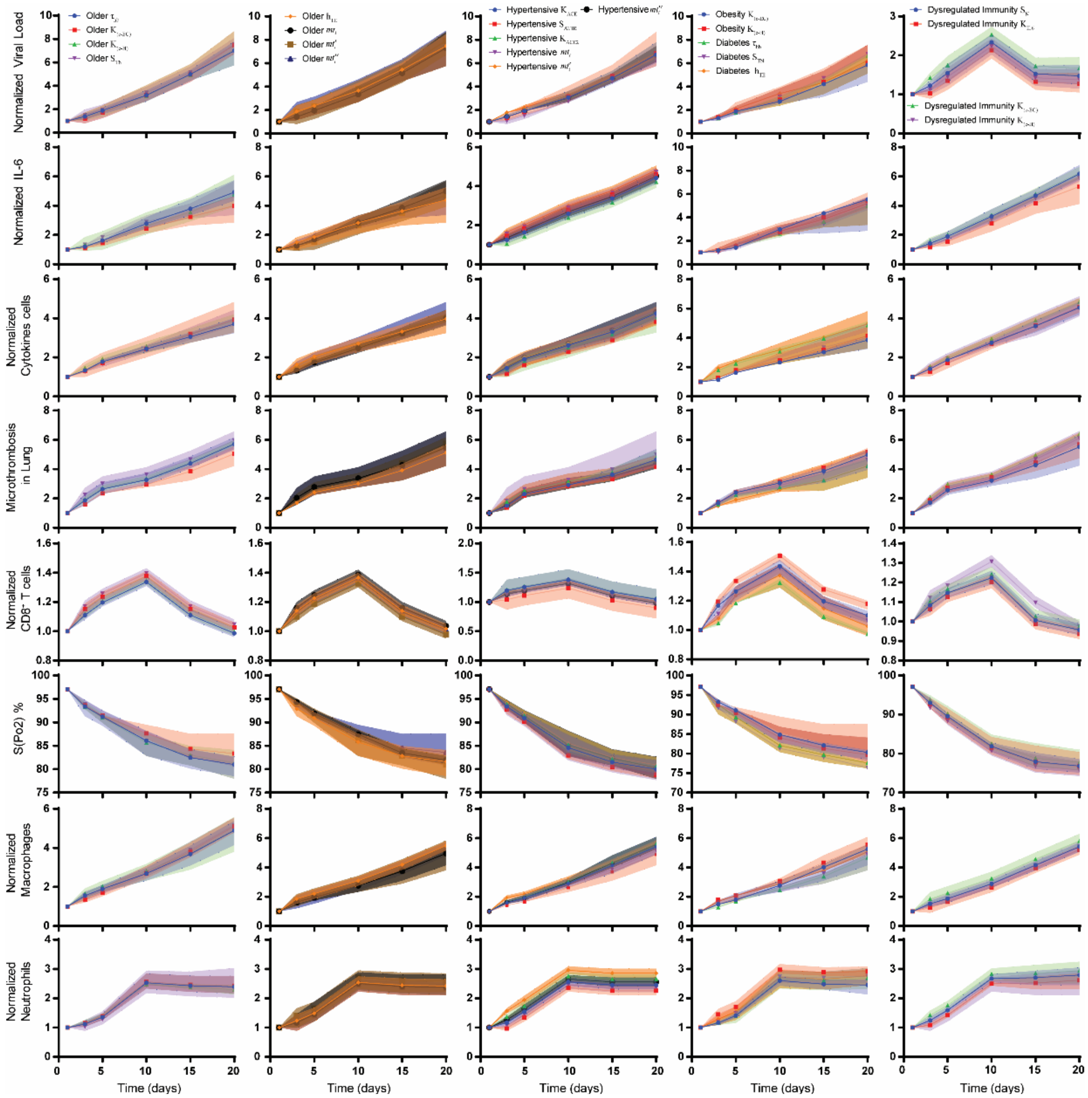

Fig. S3. Sensitivity analyses for older male patient (>65 years old) and male patients with co-morbidities: hypertension, obesity and diabetes, as well as for hyper-inflamed patients. For the analysis, we varied each parameter involved in the various patient phenotypes (Supplemental Table S2) separately by a factor of 10, 100 and 1000. The plots depict the range of values of the model variables.

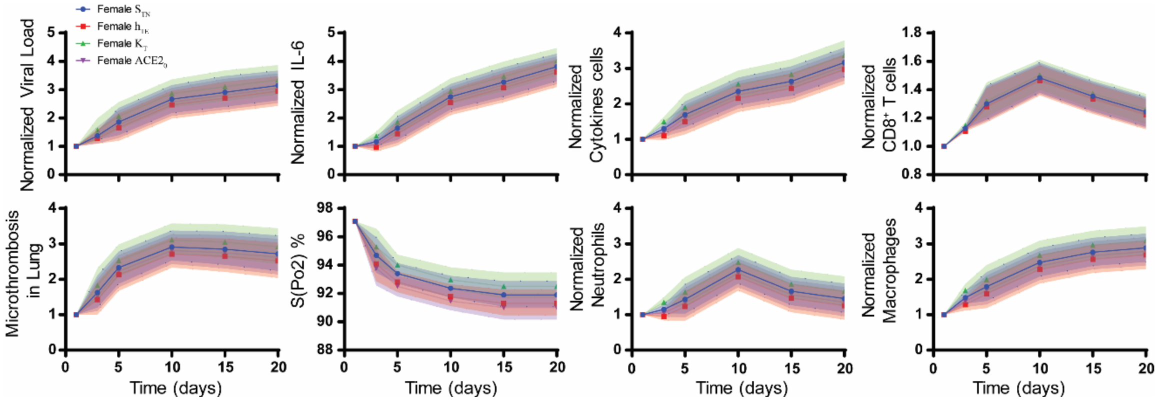

Fig. S4. Sensitivity analyses for older Female patient (>65 years old). For the analysis, we varied each parameter involved in the Female patient phenotype (Supplemental Table S2) separately by a factor of 10, 100 and 1000. The plots depict the range of values of the model variables.

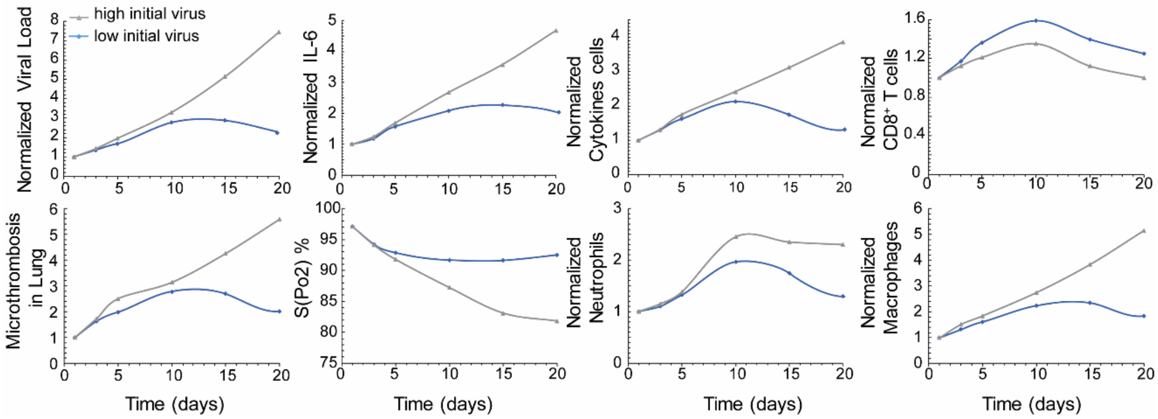

Fig S5. Model predictions for a 65 year old male for low and high initial virus load. High levels of initial viremia lead to progressive infection, whereas low levels to clearance. High initial virus corresponds to the baseline initial concentration of the virus, whereas for the low initial virus case, we reduced the initial concentration of the virus by a factor of 1000.

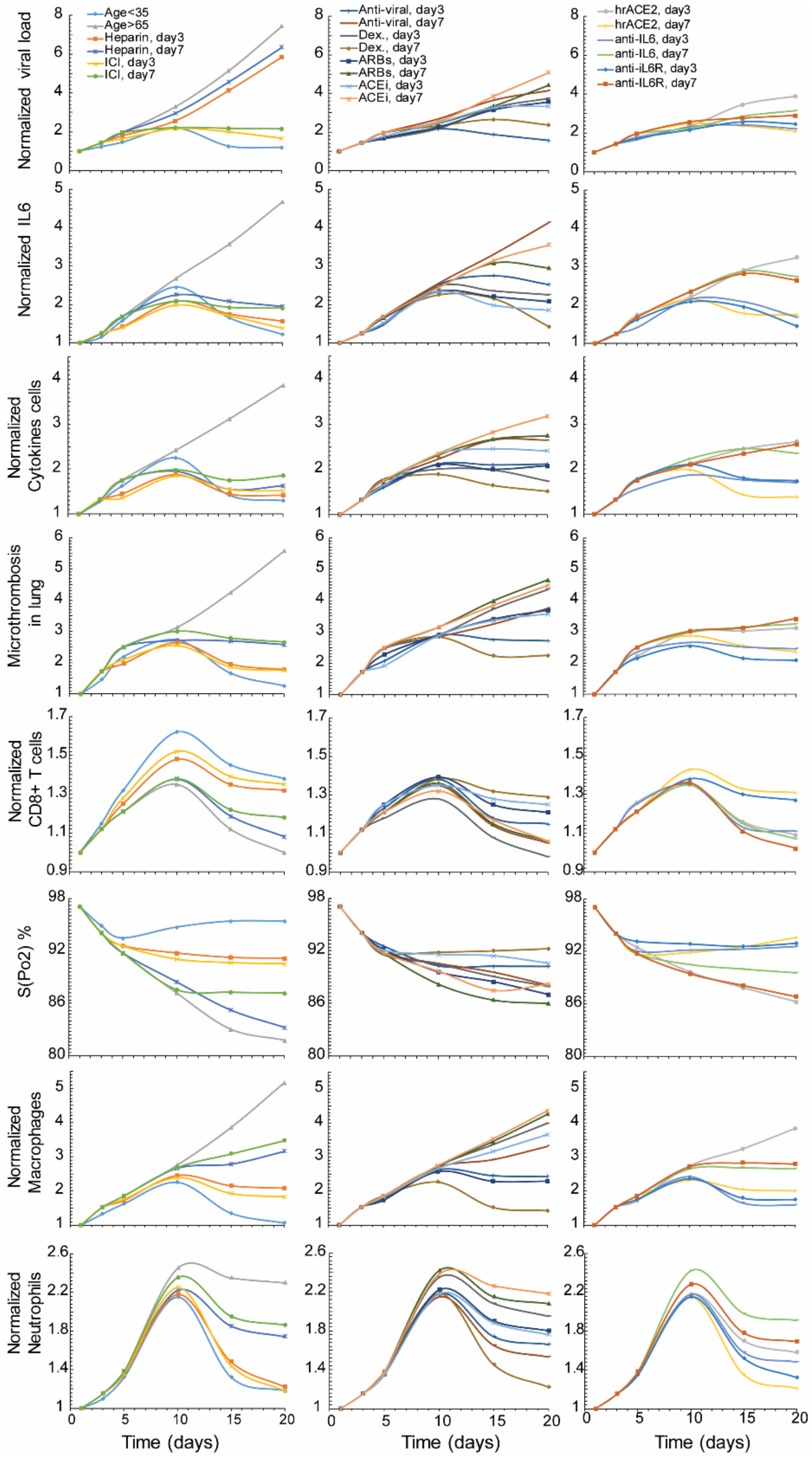

Fig. S6. Model predictions for the treatment of an at-risk (age>65) male patient using various treatments, initiated at day 3 or 7. Baseline simulations for male patients below 35 and above 65 years old are also shown for comparison. Values have been normalized to the corresponding initial values except for SpO_2.

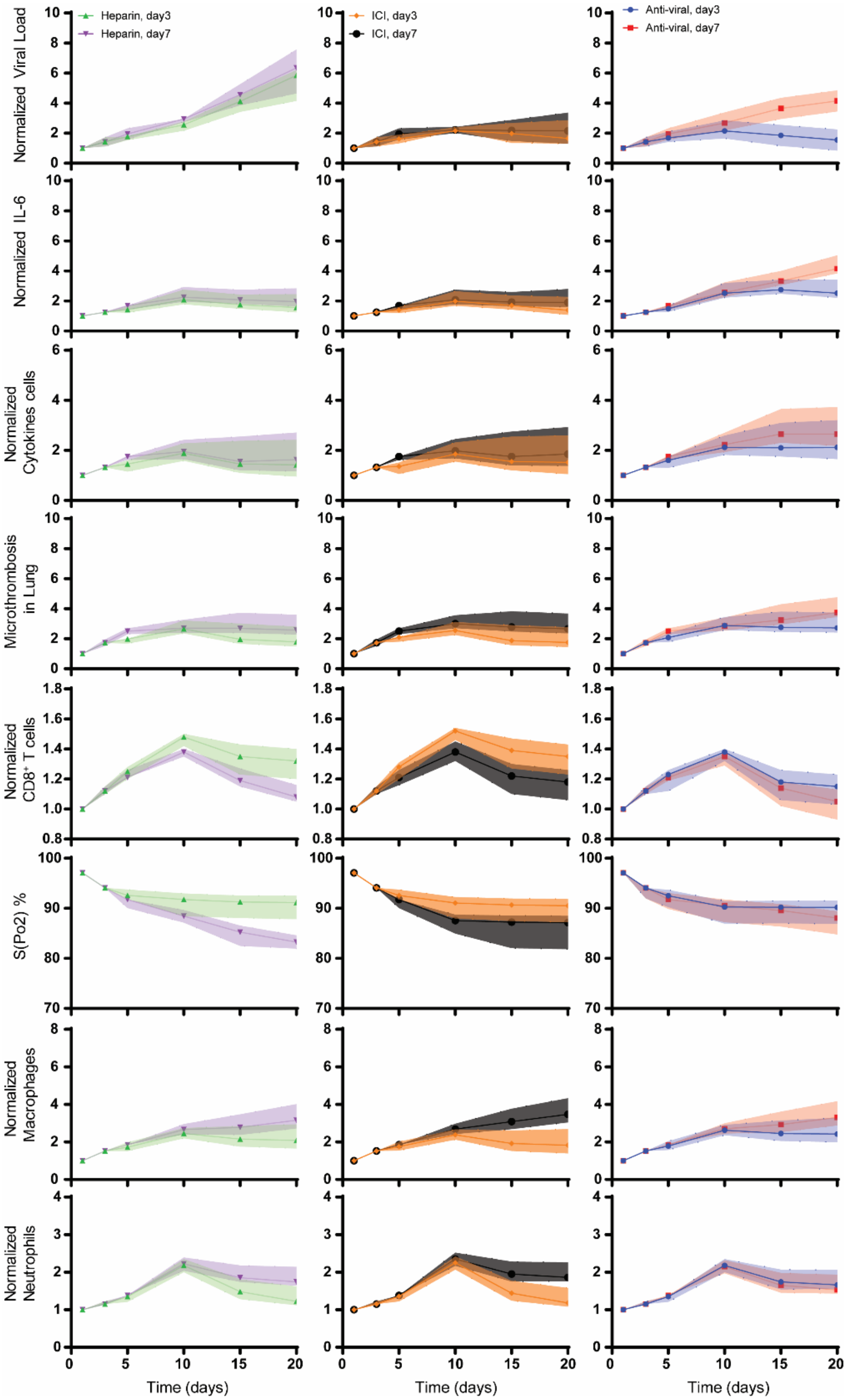

Fig. S7. Sensitivity analyses for the treatment of an at-risk (age>65) male patient using various treatments (Heparin, ICI and anti-viral), initiated at day 3 or 7. For the analysis, we varied simultaneously the values of all parameters involved in the various treatments (Supplemental Table 2) by a factor of 10, 100 and 1000. The plots depict the range of values of the model variables.

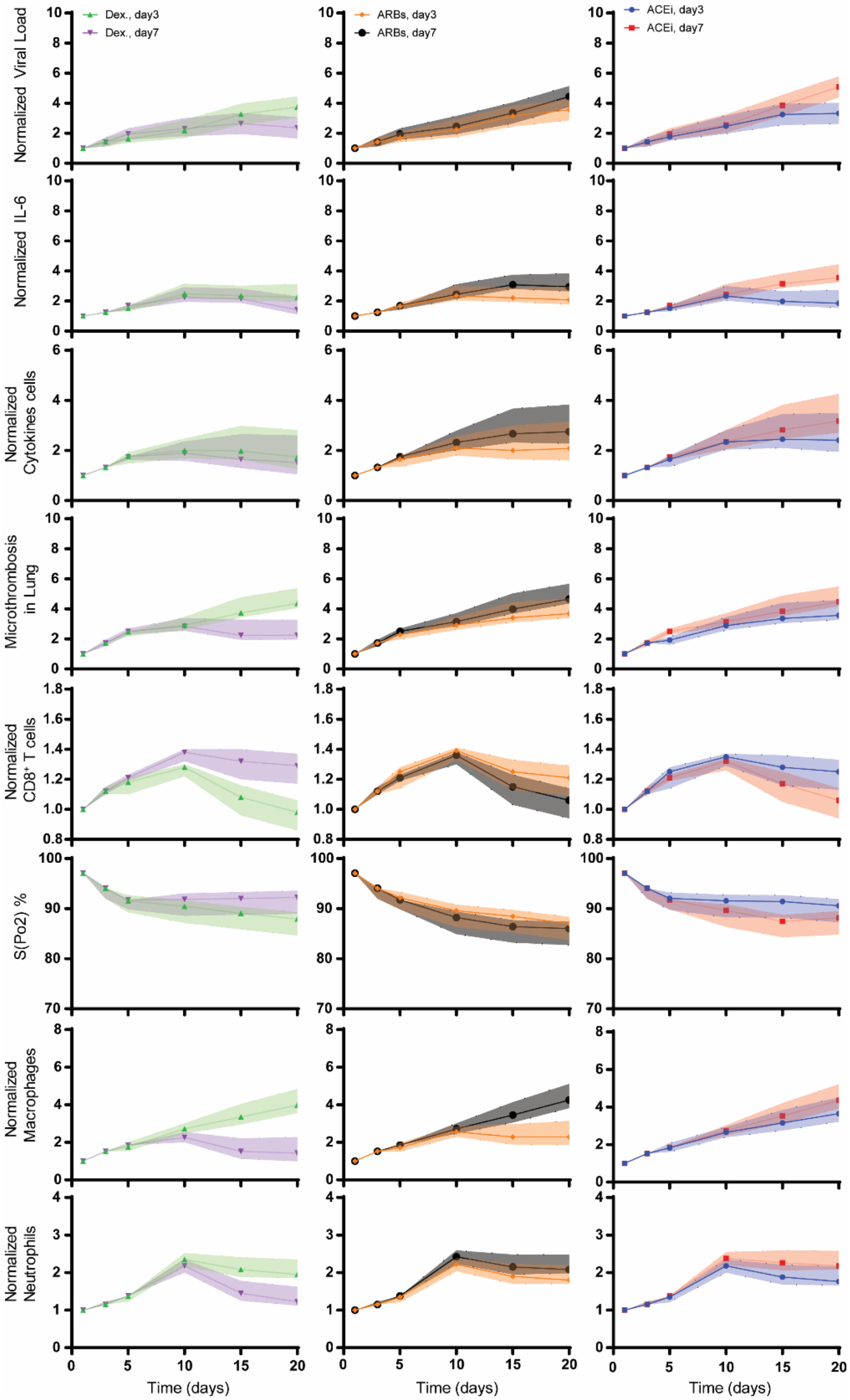

Fig. S8. Sensitivity analyses for the treatment of an at-risk (age>65) male patient using various treatments (dexamethasone, ARBs and ACEi), initiated at day 3 or 7. For the analysis, we varied simultaneously the values of all parameters involved in the various treatments (Supplemental Table 2) by a factor of 10, 100 and 1000. The plots depict the range of values of the model variables.

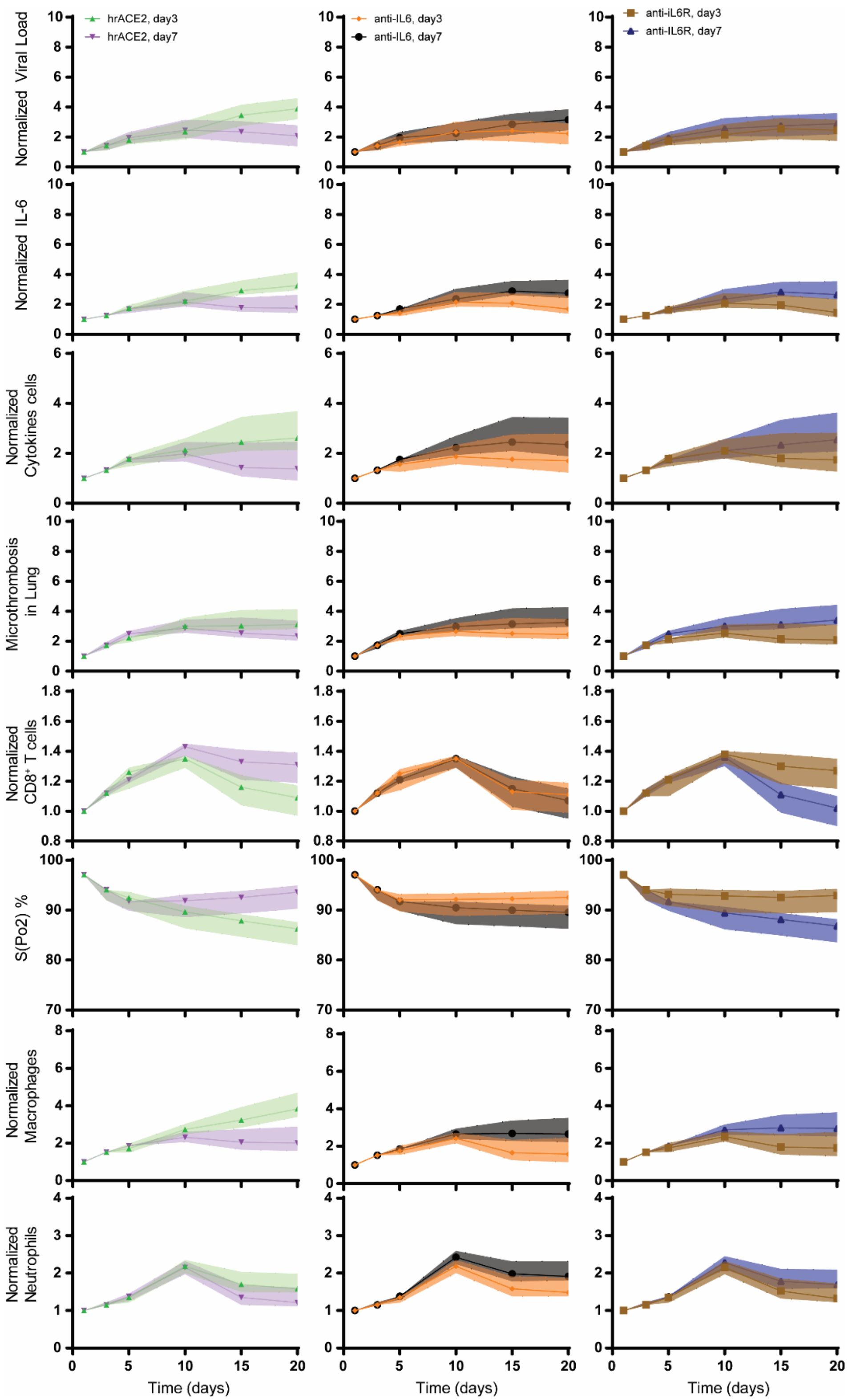

Fig. S9. Sensitivity analyses for the treatment of an at-risk (age>65) male patient using various treatments (hrACE2, anti-IL6 and IL6R), initiated at day 3 or 7. For the analysis, we varied simultaneously the values of all parameters involved in the various treatments (Supplemental Table 2) by a factor of 10, 100 and 1000. The plots depict the range of values of the model variables.

Table S1. Values of model parameters.

Parameter	Description	Value [Units]	Reference
K_{AGT}	Angiotensinogen production rate	2.27×10^{6} [nmol/L/h]	Pilvankar et al. (6)
c_{Renin}	Renin rate constant	1.8×10^{-14} [1/s]	Pilvankar et al. (7)
h_{AGT}	Angiotensinogen half-life	10 [h]	Pilvankar et al. (6)
h_{Renin}	Renin half-life	0.25 [h]	Pilvankar et al. (6)
K_{f}	Parameter for ANGII-renin feedback	4.91×10^{-5} [1/h]	Pilvankar et al. (6)
f	Parameter for ANGII-renin feedback	0.51 [nmol/ml]	Pilvankar et al. (6)
K_{Renin}	Ang I production rate constant	6.44×10^{4} [1/h]	Pilvankar et al. (6)
K_{ACE}	Rate of conversion of ANGI->ANGII	185.22[1/h]	Pilvankar et al. (6)
K_{NEP}	Rate of conversion of ANGI->ANG(1-7)	0.583 [1/h]	Pilvankar et al. (6)
h_{ANGI}	Half-life of ANGI	1.72×10^{-4} [h]	Pilvankar et al. (6)
K_{APA}	Rate of conversion of ANGII->ANGIII	43.6 [1/h]	Pilvankar et al. (6)
h_{ANGII}	Half-life of ANGII	5×10^{-3} [h]	Pilvankar et al. (6)
$h_{AT1R-ANGII}$	Half-life of AT1R bound to ANGII	1.5 [min]	Pilvankar et al. (7)
$h_{AT2R-ANGII}$	Half-life of AT2R bound to ANGII	1.5 [min]	Pilvankar et al. (7)
$h_{ANG_{(1-7)}}$	Half-life of ANG(1-7)	30 [min]	Pilvankar et al. (7)
$h_{MAsR-ANG_{(1-7}}$	Half-life of Ang(1-7) bound to MAsR	1.5 [min]	Estimate – same as $h_{(AT1R\text{-}ANGII)}$
$h_{ANG_{(1-9)}}$	Half-life of ANG(1-9)	24 [min]	Pilvankar et al. (7)
K_{APM}	Rate of conversion of ANGIII->ANGIV	43.6 [1/h]	Estimate – same as K_{APA}
h_{ANGIII}	Half-life of ANGIII	30 [s]	Pilvankar et al. (7)
h_{ANGIV}	Half-life of ANGIV	0.5 [min]	Lo A. et al. (8)
$h_{AT4R-ANGIV}$	Half-life for ANGIV bound to AT4R	1.5 [min]	Estimate – same as $h_{(AT1R\text{-}ANGII)}$
D	Diffusion of the virus in the lung	5×10^{-3} [cm^2/s]	Estimate – Mok W. et al. (14)
K_{d}	Inactivating rate of the virus	4.8×10^{-5} [1/s]	Mok W. et al. (14)
K_{a}	Rate of virus replication and release from the cell	5.78×10^{2} [1/s]	Estimate - Mok W. et al. (14)
K_{IF}	Strength of virus replication inhibition by Interferon	0.0025[pg/ml]	Estimate
K_{int}	Rate of bound virus internalization	5.78×10^{-4} [1/s]	Mok W. et al. (14)
K_{AT1R}	Rate of production of pro-inflammatory cytokines	4.2×10^{2} [pg/h/fmol]	Estimate
S_{n}	Cytokine production by innate immune cells and infected cells	2.1×10^{-2} [pg/h]	Smith A.M. et al (15)
S_{c}	Cytokine production by the internalized virus	2.9×10^{-2} [pg/ml/h]	Smith A.M. et al (15)
d_{c}	Degradation rate of pro-inflammatory cytokines	8.3×10^{-1} [1/h]	Smith A.M. et al (15)
K_{g}	Production of anti-inflammatory cytokines by macrophages and neutrophils	2.1×10^{-2} [pg/h]	Dunster J.L. et al. (10)
φ_{a}	Production of anti-inflammatory cytokines by macrophages interaction neutrophils	2.1×10^{-6} [ml]	Dunster J.L. et al. (10)
K_{Ang1-7}	Production rate of anti-inflammatory cytokines by ANG(1-7) bound to MAs receptor	4.2×10^{2} [pg/h/fmol]	Estimate
γ_{a}	Degradation rate of anti-inflammatory cytokines	3 [1/day]	Dunster J.L. et al. (10)
K_{b}	Rate of conversion of healthy to infected epithelial cells	1.36×10^{5} [1/M/s]	Su Z. and Wu Y. (16)
φ_{c}	Production of infected epithelial cells by neutrophils and pro/anti-inflammatory cytokines	2.3×10^{-9} [ml/h]	Estimate
φ_{CTL}	Rate of killing by activated T cells	2.1×10^{-10} [ml/h]	Estimate
R_{H}	Rate of production of healthy epithelial cells	2.75×10^{-3} [1/h]	Mahasa KJ et al.(9)
χ_{n}	Production rate of neutrophils by pro-inflammatory cytokines	2.1×10^{-2} [1/h]	Estimate- Dunster J.L. et al. (10)
γ_{n}	Rate of production of neutrophil NETs	6.3×10^{-4} [1/h]	Dunster J.L. et al. (10)
γ_{NETS}	Degradation rate of NETs	6.3×10^{-7}[1/h]	Estimate
χ_{m}	Production rate of macrophages by pro-inflammatory cytokines	0.02 [1/pg/h]	Estimate- Dunster J.L. et al. (10)
γ_{Ma}	Degradation rate of macrophages	6.3×10^{-4} [1/h]	Dunster J.L. et al. (10)
$K^{on}_{ACE2-ANGI}$	Rate of ANGI binding to ACE2 receptors	25 [ml/h/nmol]	Estimate – Mok W. et al. (14)
$K^{off}_{ACE2-ANGI}$	Rate of ANGI detachment from ACE2 receptors	5.22[1/h]	Estimate – Mok W. et al. (14)

Parameter	Description	Value [Units]	Reference
K_{AT1}^{on}	Rate of ANGII binding to AT1R receptors	25 [ml/h/nmol]	Estimate – Mok W. et al. (14)
K_{AT1}^{off}	Rate of ANGII detachment from AT1R receptors	5.22[1/h]	Estimate – Mok W. et al. (14)
K_{AT2}^{on}	Rate of ANGII binding to AT2R receptors	25 [ml/h/nmol]	Estimate – Mok W. et al. (14)
K_{AT2}^{off}	Rate of ANGII detachment from AT2R receptors	5.22[1/h]	Estimate – Mok W. et al. (14)
$K_{ACE2-ANGII}^{on}$	Rate of ANGII binding to ACE2 receptors	25 [ml/h/nmol]	Estimate – Mok W. et al. (14)
$K_{ACE2-ANGII}^{off}$	Rate of ANGII detachment from ACE2 receptors	15.66[1/h]	Estimate – Mok W. et al. (14)
$K_{ANG(1-9)}$	Production of ANG(1-7) from ANG(1-9)	5.22[1/h]	Estimate – Mok W. et al. (14)
K_{MAs}^{on}	Rate of ANG(1-7) binding to MAs receptors	25 [ml/h/nmol]	Estimate – Mok W. et al. (14)
K_{MAs}^{off}	Rate of ANG(1-7) detachment from MAS receptors	5.22[1/h]	Estimate – Mok W. et al. (14)
K_{AT4}^{on}	Rate of ANGIV binding to AT4R receptors	25 [ml/h/nmol]	Estimate – Mok W. et al. (14)
K_{AT4}^{off}	Rate of ANGIV detachment from AT4R receptors	5.22[1/h]	Estimate – Mok W. et al. (14)
K_{IL6}	Production rate of IL6 by activated macrophages	0.5 [ml/h]	Estimate
γ_{IL6}	Degradation rate of IL6	6.3 x10^{-4} [1/h]	Estimate
K_{IL6}^{on}	Rate of IL6 binding to IL6 receptors	15 [ml/h/nmol]	Estimate – Mok W. et al. (14)
K_{IL6}^{off}	Rate of IL6 detachment from IL6 receptors	5.22[1/h]	Estimate – Mok W. et al. (14)
h_{IL6R}	The half-life for IL6 receptor	1.5 [min]	Estimate – same as $h_{AT1R-ANGII}$
S_{sIL6R}	Source production of sIL6R	0.57[fmol/ml/h]	Estimate – same as s_{Renin}
K_{sIL6R}^{on}	Rate of IL6 binding to soluble IL6 receptors	17 [ml/h/nmol]	Estimate – Mok W. et al. (14)
S_{VEGF}	Source production of VEGF	0.235[fmol/ml/h]	Estimate – same as $\frac{s_{Renin}}{2}$
K_{VEGF}	Production rate of VEGF by IL6 bound on the soluble IL6 receptor	5.22[1/h]	Estimate – Mok W. et al. (14)
$K_{AT1R-MAsR}$	Degradation rate of pro-inflammatory cytokines by Mas receptor	5.22[1/h]	Estimate – Mok W. et al. (14)
$K_{AT1R-AT2R}$	Degradation rate of pro-inflammatory cytokines by AT2 receptor	5.22[1/h]	Estimate – Mok W. et al. (14)
S_{AT1R}	Source term for AT1R	0.57[fmol/ml/h]	Estimate – same as s_{Renin}
S_{AT2R}	Source production of AT2R	0.57[fmol/ml/h]	Estimate – same as s_{Renin}
S_{MAsR}	Source production of MAsR	0.57[fmol/ml/h]	Estimate – same as s_{Renin}
S_{AT4R}	Source production of AT4R	0.57[fmol/ml/h]	Estimate – same as s_{Renin}
S_{IL6R}	Source production of IL6R receptor	0.57[fmol/ml/h]	Estimate – same as s_{Renin}
K_{sIL6R}	Conversion rate of IL6 receptor to soluble IL6 receptor	5.22[1/h]	Estimate – Mok W. et al. (14)
K_{sIL6R}^{off}	Rate of IL6 detachment from soluble IL6 receptors	2.22[1/h]	Estimate – Mok W. et al. (14)
S_{sACE2}	Source production of sACE2	0.57[fmol/ml/h]	Estimate – Mok W. et al. (14)
K_{sACE2}	Rate of soluble ACE2 receptor binding to the virus	17 [ml/h/nmol]	Estimate – Mok W. et al. (14)
K_{Adam17}	Production rate of soluble ACE2 receptor by ACE2 receptor interaction through Adam17	5.22[1/h]	Estimate – Mok W. et al. (14)
$K_{ACE2-Virus}^{on}$	Rate of virus binding to ACE2 receptors	17 [ml/h/nmol]	Estimate – Mok W. et al. (14)
$K_{ACE2-Virus}^{off}$	Rate of virus detachment from ACE2 receptors	5.22[1/h]	Estimate – Mok W. et al. (14)
χ_{N-IL6}	Production of neutrophils by IL6-R activation	5.26[1/fmol/h]	Estimate- Dunster J.L. et al. (10)
χ_{Ma-IL6}	Production of macrophages by IL6-R activation	5.26[1/fmol/h]	Estimate- Dunster J.L. et al. (10)
K_{ec}	Proliferation of endothelial cells	5.22x10^{-7} [1/h]	Estimate
K_{ec}^{b}	Endothelial cell infection rate	70 [ml/h/nmol]	Estimate
S_{v}^{0}	Vascular density of the normal lung	70 [1/cm]	Mpekris F. et al (17)
K_{ACE2}	Production of ANG(1-7) by ANGII bound to ACE2	65.22[1/h]	Pilvankar et al. (6)
S_{ACE2}	Production rate of ACE2 by healthy endothelial and epithelial cells	0.57[fmol/ml/h]	Estimate – same as s_{Renin}
V_{O2}^{max}	oxygen uptake rate	3700 [mlO_2/min]	Weibel E.R. et al (11)
P_{A}	Partial pressure of oxygen in alveolar air	100 [mmHg]	T.K. Roy, T.W. Secomb (18)
K_{02}^{0}	Krogh permeability coefficient KO2	3.3x10^{-8} [cm^2/min/mmHg]	Weibel E.R. et al (11)

Parameter	Description	Value [Units]	Reference
$S(A)$	Alveolar gas exchange areas	130 [m^2]	Weibel E.R. et al (11)
$S(c)$	Capillary gas exchange areas	115 [m^2]	Weibel E.R. et al (11)
τ_{hb}	Harmonic mean thickness of the air–blood barrier	1 [μm]	Weibel E.R. et al (11)
θ_{O2}	Oxygen unloading conductance of blood	1.8[mlO_2/ml/min/mmHg]	Roy T.K., Secomb T.W. (18)
$V(c)$	Lung blood volume	194 [ml]	Roy T.K., Secomb T.W. (18)
n	Hill coefficient	2.7	Roy T.K., Secomb T.W. (18)
P_{50}	Oxygen tension when the binding sites are 50 percent saturated.	26.3 [mmHg]	Roy T.K., Secomb T.W. (18)
K_{in}	Rate of release of replicated virus	0.4 x10^{-7} [1/h]	Mahasa KJ et al.(9)
d_{AT1R}	Degradation rate of AT1R	6.3x10^{-4}[1/h]	Estimate- Dunster J.L. et al. (10)
d_{AT2R}	Degradation rate of AT2R	6.3x10^{-4}[1/h]	Estimate- Dunster J.L. et al. (10)
d_{MAsR}	Degradation rate of MAsR	6.3x10^{-4}[1/h]	Estimate- Dunster J.L. et al. (10)
d_{AT4R}	Degradation rate of AT4R	6.3x10^{-4}[1/h]	Estimate- Dunster J.L. et al. (10)
d_{IL6R}	Degradation rate of IL6R	6.3x10^{-4}[1/h]	Estimate- Dunster J.L. et al. (10)
d_{sACE2}	Degradation rate of soluble ACE2 receptor	6.3x10^{-4}[1/h]	Estimate- Dunster J.L. et al. (10)
K_{IL6-TN}	Production rate of IL6 by Naïve T cells	0.02554[mol/pg/s]	Estimate - Lai, X., & Friedman A. (12)
K_{IL6-In}	Production rate of IL6 by infected epithelial cells	0.02554[mol/pg/s]	Estimate - Lai, X., & Friedman A. (12)
K_{IL6-TE}	Production rate of IL6 by Activated T cells	0.02554[mol/pg/s]	Estimate - Lai, X., & Friedman A. (12)
$K_{IL6-iEC}$	Production rate of IL6 by infected endothelial cells	0.02554[mol/pg/s]	Estimate - Lai, X., & Friedman A. (12)
γ_{VEGF}	Production rate of VEGF by hypoxia	0.0152[mol/ml/h]	Estimate
K_{AT1R}	Production of pro-inflammatory cytokines by AT1R activation	4.2x10^2 [pg/h/fmol]	Estimate
K_{c-IL6}	Production of pro-inflammatory cytokines by IL6-R activation	0.03 [ml/h/fmol]	Estimate
K_{c-EC}	Production of pro-inflammatory cytokines by healthy endothelial cells	1 [pg]	Estimate
K_{c-H}	Production of pro-inflammatory cytokines by healthy epithelial cells	1 [pg]	Estimate
K_H	Parameter for production of infected epithelial cells by neutrophils and pro/anti-inflammatory cytokines	0.02554 [ml/pg]	Estimate
S_{TN}	Source production of naïve T cells	0.235 [pg/ml/h]	Estimate - Lai, X., & Friedman A. (12)
h_{TE}	Conversion rate of Naïve T cells to activated T cells	0.00254 [1/h]	Estimate - Lai, X., & Friedman A. (12)
As	Antigen strength	1	Estimate - Lai, X., & Friedman A. (12)
K_T	Constant for blocking PD-1 inhibition	1.365x10^{-18} [g/cm^3]	Estimate - Lai, X., & Friedman A. (12)
ε	Degradation of activated T cells by PD1 bound to PDL1	0.01575 [cm^3/g/s]	Estimate - Lai, X., & Friedman A. (12)
d_H	Degradation rate PDL1 by healthy epithelial cells	0.00215 [1/s]	Estimate - Lai, X., & Friedman A. (12)
d_{EC}	Degradation rate PDL1 by endothelial healthy cells	0.00215 [1/s]	Estimate - Lai, X., & Friedman A. (12)
d_{In}	Degradation rate PDL1 by epithelial infected Cells	0.00215 [1/s]	Estimate - Lai, X., & Friedman A. (12)
d_{TE}	Degradation rate PD1 by Activated T cells	0.00215x10^{-3} [1/s]	Estimate - Lai, X., & Friedman A. (12)
d_{TN}	Degradation rate PD1 by Naïve T cells	0.00215 [1/s]	Estimate - Lai, X., & Friedman A. (12)
d_N	Degradation rate PD1 by neutrophils	0.00215 [1/s]	Estimate - Lai, X., & Friedman A. (12)
d_{Ma}	Degradation rate PD1 by macrophages	0.00215 [1/s]	Estimate - Lai, X., & Friedman A. (12)
a_{PL}	Association of PD-1 with PD-L1	0.258[mm^3/g/s]	Estimate - Lai, X., & Friedman A. (12)
d_Q	Dissociation rate of PD-L1 from PD-1	0.1 [1/d]	Estimate - Lai, X., & Friedman A. (12)
γ_A	Source of anti-PD1	1x10^{-10}[g/cm^3/d]	Estimate - Lai, X., & Friedman A. (12)
μ_{PD1}	Efficiency of PD1 blocking by ICI	0.00215x10^{-3}[1/s]	Estimate - Lai, X., & Friedman A. (12)
d_A	Degradation rate of anti-PD1	0.0462 [1/d]	Estimate - Lai, X., & Friedman A. (12)
λ_H	Production rate of PDL1 by healthy epithelial cells	0.154 [1/s]	Estimate - Lai, X., & Friedman A. (12)
λ_{EC}	Production rate of PDL1 by healthy endothelial cells	0.154 [1/s]	Estimate - Lai, X., & Friedman A. (12)
λ_{In}	Production rate of PDL1 by infected endothelial cells	0.154 [1/s]	Estimate - Lai, X., & Friedman A. (12)
λ_{TE}	Production rate of PD1 by activated T cells	0.154 [1/s]	Estimate - Lai, X., & Friedman A. (12)
λ_{TN}	Production rate of PD1 by Naïve T cells	0.154 [1/s]	Estimate - Lai, X., & Friedman A. (12)
λ_N	Production rate of PD1 by neutrophils	0.154 [1/s]	Estimate - Lai, X., & Friedman A. (12)

Parameter	Description	Value [Units]	Reference
λ_{Ma}	Production rate of PD1 by macrophages	0.154 [1/s]	Estimate - Lai, X., & Friedman A. (12)
h_{PDL1}	Production rate of the PD1 ligand by Effector (Activated) T	0.154 [1/s]	Estimate - Lai, X., & Friedman A. (12)
μ_{PD1}	Degradation rate of PD1 by anti-PD1	0.00215 x10^{-3} [1/s]	Estimate - Lai, X., & Friedman A. (12)
a_{PL}	rate of PD1 binding to PDL1	0.258 [mm^3/g/s]	Estimate - Lai, X., & Friedman A. (12)
γ_A	Source term of anti-PD1	1x10^{-10} [g/cm^3/d]	Estimate - Lai, X., & Friedman A. (12)
μ_A	Degradation rate of anti-PD1 by PD1	6.87x10^6 [cm^3/g/d]	Estimate - Lai, X., & Friedman A. (12)
Q_{liver}	Blood flow rate of liver (hepatic portal vein from G.I. and spleen, and hepatic artery)	800 [ml/min]	(19)
Q_{spleen}	Blood flow rate of spleen	138 [ml/min]	(19)
$Q_{G.I.}$	Blood flow rate of G.I.	468 [ml/min]	(19)
Q_{kidney}	Blood flow rate of kidney	630 [ml/min]	(19)
Q_{Torso}	Blood flow rate of Torso	220 [ml/min]	Estimate - (19)
$Q_{lower\ body}$	Blood flow rate of lower body	413 [ml/min]	Estimate - (19)
$Q_{uppe\ body}$	Blood flow rate of upper body	138 [ml/min]	Estimate - (19)
Q_{brain}	Blood flow rate of brain	300 [ml/min]	Estimate - (19)
$Q_{cardic\ vessels}$	Blood flow rate of cardiac vessels	120 [ml/min]	Estimate - (19)
L_{liver}	Lymphatic flow rate of liver	8.7x10^{-2} [ml/min]	(19)
L_{spleen}	Lymphatic flow rate of spleen	8.7x10^{-4} [ml/min]	(19)
$L_{G.I.}$	Lymphatic flow rate of G.I.	3.0 x 10^{-1} [ml/min]	(19)
L_{kidney}	Lymphatic flow rate of kidney	1x 10^{-3} [ml/min]	Estimate - (19)
L_{Torso}	Lymphatic flow rate of torso	4.3x 10^{-3} [ml/min]	Estimate - (19)
$L_{lower\ body}$	Lymphatic flow rate of lower body	1x 10^{-3} [ml/min]	Estimate - (19)
$L_{uppe\ body}$	Lymphatic flow rate of upper body	2.6 x 10^{-2} [ml/min]	Estimate - (19)
L_{brain}	Lymphatic flow rate of brain	1x 10^{-3} [ml/min]	Estimate - (19)
$L_{cardic\ vessels}$	Lymphatic flow rate of cardiac vessels	4.3x 10^{-3} [ml/min]	Estimate - (19)
L_{lung}	Lymphatic flow rate of lung	4.3x 10^{-2} [ml/min]	(19)
V_{liver}	Averaged vascular volume of liver	180.9 [ml]	(19)
V_{spleen}	Averaged vascular volume of spleen	17 [ml]	(19)
$V_{G.I.}$	Averaged vascular volume of GI system	43 [ml]	(19)
V_{kidney}	Averaged vascular volume of kidney	28.4 [ml]	(19)
V_{Torso}	Averaged vascular volume of torso	462 [ml]	Estimate
$V_{lower\ body}$	Averaged vascular volume of lower body	700 [ml]	Estimate
$V_{uppe\ body}$	Averaged vascular volume of upper body	150 [ml]	Estimate
V_{brain}	Averaged vascular volume of brain	150 [ml]	Estimate
$V_{cardic\ vessels}$	Averaged vascular volume of cardiac vessels	100 [ml]	Estimate
V_{lung}	Averaged vascular volume of lung	99.9 [ml]	(19)

Parameter	Description	Value [Units]	Reference
a_i	Attachment rate of virus to ACE2	1x 10^{-3} [1/(mM.min)]	Estimate
a'	Attachment rate of virus to sACE2	8.5 [ml/h/nmol]	Estimate
d_i	Detachment rate of virus from endothelium	6.7x 10^{-4} [1/min]	Estimate
mt_i	Maximum production of micro-thrombus (ACE2)	7x 10^{-3} [mM/min]	Estimate
mt_i'	Maximum production of micro-thrombus (IL6)	7x 10^{-3} [mM/min]	Estimate
mt_i''	Maximum production of micro-thrombus (cytokines)	7x 10^{-3} [mM/min]	Estimate
mt'''	Maximum production of micro-thrombus (NET)	7x 10^{-3} [mM/min]	Estimate
ε_i	Dissolution of micro-thrombi due to thrombolysis	7x 10^{-5} [1/min]	Estimate
dm_{liver}	Attachment rate of micro-thrombus to vessels in liver	6.9x 10^{-6} [1/min]	Estimate
dm_i	Attachment rate of micro-thrombus to vessels in other organs	6.9x 10^{-6} [1/min]	Estimate
p_{lung}	Proliferation rate of internalized virus in lung	7x 10^{-3} [1/min]	Estimate
p_{liver}	Proliferation rate of internalized virus in liver	7x 10^{-3} [1/min]	Estimate
p_{spleen}	Proliferation rate of internalized virus in spleen	0.7x 10^{-3} [1/min]	Estimate
$p_{\text{upper body}}$	Proliferation rate of internalized virus in upper body	7.6x 10^{-3} [1/min]	Estimate
p_{Torso}	Proliferation rate of internalized virus in torso	14x 10^{-3} [1/min]	Estimate
$p_{\text{lower body}}$	Proliferation rate of internalized virus in lower body	6.3x 10^{-3} [1/min]	Estimate
$p_{\text{intestine}}$	Proliferation rate of internalized virus in intestine	4.9x 10^{-3} [1/min]	Estimate
p_{brain}	Proliferation rate of internalized virus in brain	9.7x 10^{-3} [1/min]	Estimate
p_{kidney}	Proliferation rate of internalized virus in kidney	7.6x 10^{-3} [1/min]	Estimate
$p_{\text{cardiac vessels}}$	Proliferation rate of internalized virus in cardiac vessels	12.5x 10^{-3} [1/min]	Estimate
k_{acg}	Micro-thrombus-inhibiting coefficient of anti-coagulation drugs for virus-ACE-2-induced coagulation	0.5	Estimate
k'_{acg}	Micro-thrombus-inhibiting coefficient of anti-coagulation drugs for IL6-induced coagulation	0.5	Estimate
k''_{acg}	Micro-thrombus-inhibiting coefficient of anti-coagulation drugs for cytokine storm-induced coagulation	0.5	Estimate
k'''_{acg}	Micro-thrombus-inhibiting coefficient of anti-coagulation drugs for NET-induced coagulation	0.5	Estimate

Initial Values (Normal Value)

Parameter	Value
$[ACE2]_0$	5.17 [fmol/ml]
$[ACE2bAngI]_0$	4.1 x 10^{-8} [mol/L]
$[ACE2bAngII]_0$	2.1 x 10^{-8} [mol/L]
$[AGT]_0$	1.7 x 10^{7} [nmol/L]
$[Ang17]_0$	1.2 x 10^{-7} [mol/L]
$[Ang19]_0$	5.9 x 10^{-8}mol/L]
$[AngI]_0$	19.6[nmol/L]
$[AngII]_0$	152[nmol/L]
$[AngIII]_0$	12.94[nmol/L]

$[AngIV]_0$	6.99[nmol/L]
$[AT1R]_0$	2.26[fmol/ml]
$[AT1R-AngII]_0$	4.1 x 10^{-8} [mol/L]
$[AT2R]_0$	1.14[fmol/ml]
$[AT2R-AngII]_0$	2.1 x 10^{-8} [mol/L]
$[AT4R]_0$	0.57[fmol/ml]
$[AT4R-AngIV]_0$	1.1 x 10^{-8} [mol/L]
c_0	4.96 x 10^{4} [pg/ml]
C_{tl0}	2.17 x 10^{9} [1/L]
$[H]_0$	7.23 x 10^{8} [1/ml]
$[IL6]_0$	16[fmol/ml]
$[IL6R]_0$	1.15[fmol/ml]
$[IL6R-IL6]_0$	2.1 x 10^{-8} [mol/L]
M_{a0}	2.17 x 10^{9} [1/L]
$[MAsR-Ang17]_0$	2.1 x 10^{-8} [mol/L]
$[MAsR]_0$	2.23[fmol/ml]
N_0	4.2 x 10^{9} [1/L]
$[Renin]_0$	2.06 x 10^{-4} [nmol/L]
$[sACE]_0$	0.03[fmol/ml]
$[sIL6R]_0$	0.03[fmol/ml]
$[sIL6R-IL6]_0$	1.05 x 10^{-8} [mol/L]
$[VEGF]_0$	3.29 x 10^{-4} [fmol/ml]
$[EC]_0$	7.23 x 10^{6} [1/ml]

Table S2. Parameter perturbations for various patient phenotypes and treatments. To simulate the listed phenotypes or treatments, the following parameter adjustments were made:

(red)	Parameter Increased
(blue)	Parameter Decreased

Phenotypes:

Parameter	Description	Older	Hyper-tensive	Dys-regulated Immunity	Obesity	Diabetes	Female
K_{ACE}	Rate of conversion of ANGI->ANGII		Increased				
S_n	Cytokine production from innate immune cells and infected cells			Increased			
K_{IL6}	Production rate of IL6 by virus activated macrophages			Increased			
S_{AT1R}	Source term for AT1R		Increased				
K_{ACE2}	Rate constant for production of ANG(1-7) by ANGII bound to ACE2		Decreased				
τ_{hb}	Harmonic mean thickness of the air–blood barrier	Increased				Increased	
K_{c-EC}	Production rate of pro-inflammatory cytokines by healthy endothelial cells	Increased		Increased	Increased		
K_{c-H}	Production rate of pro-inflammatory cytokines by healthy epithelial cells	Increased		Increased	Increased		
S_{TN}	Source production of naïve T cells	Decreased				Decreased	Decreased
h_{TE}	Conversion rate of Naïve T cells to activated T cells	Decreased				Decreased	Decreased
As	Antigen strength						
K_T	Constant for blocking PD-1 inhibition						Decreased
mt_i	Maximum production rates of micro-thrombus (ACE2)	Increased	Decreased				
mt_i'	Maximum production rates of micro-thrombus (IL6)	Increased	Decreased				
mt_i''	Maximum production rates of micro-thrombus (cytokines)	Increased	Decreased				
$ACE2_0$	Initial ACE2 concentration						Decreased

Treatments:

Parameter	Description	Heparin	ICI	Anti-viral	Dex.	ARBs	ACEi	hrACE2	Anti-IL6	Anti-IL6R
K_{ACE}	Rate of conversion of ANGI->ANGII						Decreased			
K_d	Inactivating rate of the virus			Increased						
S_n	Cytokine production from innate immune cells and infected cells				Decreased					
K_{AT1}^{on}	Rate of binding ANGII on the AT1R receptors					Decreased				
K_{AT1}^{off}	Rate of unbinding ANGII on the AT1R receptors					Increased				
K_{IL6}	Production rate of IL6 by virus activated macrophages				Decreased				Decreased	
S_{IL6R}	Source production of IL6R receptor									Decreased

K_{ACE2}	Rate constant for production of ANG(1-7) by ANGII bound to ACE2									
K_{in}	Rate of release of replicated virus									
K_{IL6-TN}	Production rate of IL6 by Naïve T cells									
K_{IL6-In}	Production rate of IL6 by infected epithelial cells									
K_{IL6-TE}	Production rate of IL6 by Activated T cells									
$K_{IL6-iEC}$	Production rate of IL6 by infected endothelial cells									
d_Q	Dissociation of PD-L1 from PD-1									
a_{PL}	Association of PD-1 with PD-L1									
γ_A	Source of anti-PD1									
a_i	Attachment rate of virus to ACE2									
d_i	Detachment rate of virus from endothelium									
mt_i	Maximum production rates of micro-thrombus (ACE2)									
mt_i'	Maximum production rates of micro-thrombus (IL6)									
mt_i''	Maximum production rates of micro-thrombus (cytokines)									
dm_{liver}	Attachment rate of micro-thrombus to vessels in liver									
dm_i	Attachment rate of micro-thrombus to vessels in other organs									

Table S3. Summary of model predictions for the therapeutic outcome of combined treatments for obese male patients (>65 years old). The table presents the results at the end of the simulation (day 20). Values have been normalized to the corresponding initial values except for S(pO2).

SpO_2 (%)	Obesity	Heparin, day3	ICI, day3	Anti-viral, day3	Dex., day7	ARBs, day3	ACEi, day3	hrACE2, day7	anti-IL6, day3	anti-iL6R, day3
Obesity	80	92	90	90	91	85	89	91	91	90
Heparin, day3			94	92	94	91	91	92	92	92
ICI, day3				91	94	92	92	92	93	93
Anti-viral, day3					92	88	91	91	91	91
Dex., day7						93	93	93	93	93
ARBs, day3							90	91	92	91
ACEi, day3								93	92	91
hrACE2, day7									93	93
anti-IL6, day3										93
anti-iL6R, day3										

Normalized T eff cells	Obesity	Heparin, day3	ICI, day3	Anti-viral, day3	Dex., day7	ARBs, day3	ACEi, day3	hrACE2, day7	anti-IL6, day3	anti-iL6R, day3
Obesity	1.12	1.35	1.42	1.21	1.25	1.18	1.21	1.33	1.15	1.22
Heparin, day3			1.52	1.38	1.42	1.35	1.36	1.45	1.39	1.4
ICI, day3				1.45	1.58	1.39	1.41	1.43	1.32	1.35
Anti-viral, day3					1.38	1.28	1.25	1.32	1.35	1.38
Dex., day7						1.45	1.38	1.38	1.42	1.45
ARBs, day3							1.24	1.26	1.25	1.23
ACEi, day3								1.24	1.28	1.23
hrACE2, day7									1.4	1.42
anti-IL6, day3										1.38
anti-iL6R, day3										

Lung Microthrombi	Obesity	Heparin, day3	ICI, day3	Anti-viral, day3	Dex., day7	ARBs, day3	ACEi, day3	hrACE2, day7	anti-IL6, day3	anti-iL6R, day3
Obesity	4.98	1.98	2.23	2.52	2.34	3.25	3.62	2.12	2.15	1.98
Heparin, day3			1.48	1.56	1.62	1.88	1.85	1.77	1.65	1.58
ICI, day3				1.58	1.69	1.82	1.78	1.62	1.58	1.73
Anti-viral, day3					2.12	2.25	2.32	2.1	1.82	1.95
Dex., day7						2.08	2.15	1.88	1.95	1.64
ARBs, day3							3.15	2.08	2.12	1.95
ACEi, day3								2.06	2.08	1.93
hrACE2, day7									1.98	1.88
anti-IL6, day3										1.72
anti-iL6R, day3										

Normalized IL6	Obesity	Heparin, day3	ICI, day3	Anti-viral, day3	Dex., day7	ARBs, day3	ACEi, day3	hrACE2, day7	anti-IL6, day3	anti-iL6R, day3
Obesity	5.54	2.15	1.86	2.25	1.95	2.56	2.77	1.98	1.66	1.65
Heparin, day3			1.42	1.48	1.42	1.45	1.52	1.55	1.32	1.25
ICI, day3				1.26	1.18	1.32	1.28	1.42	1.25	1.22
Anti-viral, day3					1.52	1.56	1.45	1.38	1.28	1.32
Dex., day7						1.42	1.38	1.25	1.22	1.18
ARBs, day3							2.22	1.84	1.65	1.54
ACEi, day3								1.72	1.52	1.56
hrACE2, day7									1.35	1.24
anti-IL6, day3										1.12
anti-iL6R, day3										

Inflammatory Cytokines	Obesity	Heparin, day3	ICI, day3	Anti-viral, day3	Dex., day7	ARBs, day3	ACEi, day3	hrACE2, day7	anti-IL6, day3	anti-iL6R, day3
Obesity	4.15	1.62	1.75	2.45	1.84	2.12	2.24	1.38	1.64	1.74
Heparin, day3			1.32	1.42	1.15	1.58	1.42	1.22	1.38	1.33
ICI, day3				1.35	1.12	1.54	1.48	1.29	1.32	1.35
Anti-viral, day3					1.52	1.75	1.68	1.32	1.58	1.63
Dex., day7						1.32	1.54	1.21	1.35	1.32
ARBs, day3							1.78	1.35	1.56	1.42
ACEi, day3								1.33	1.42	1.58
hrACE2, day7									1.28	1.23
anti-IL6, day3										1.32
anti-iL6R, day3										

Normalized Viral Load	Obesity	Heparin, day3	ICI, day3	Anti-viral, day3	Dex., day7	ARBs, day3	ACEi, day3	hrACE2, day7	anti-IL6, day3	anti-iL6R, day3
Obesity	5.98	5.12	2.22	1.83	2.42	2.57	2.95	2.62	2.35	2.21
Heparin, day3			1.56	1.42	2.15	2.2	2.18	1.95	1.93	1.88
ICI, day3				1.32	1.18	1.58	1.62	1.68	1.53	1.48
Anti-viral, day3					1.06	1.41	1.45	1.55	1.43	1.32
Dex., day7						1.84	1.65	1.76	1.74	1.45
ARBs, day3							2.54	2.48	2.11	1.95
ACEi, day3								2.25	1.98	2.13
hrACE2, day7									1.47	1.52
anti-IL6, day3										1.35
anti-iL6R, day3										

Normalized Neutrophils	Obesity	Heparin, day3	ICI, day3	Anti-viral, day3	Dex., day7	ARBs, day3	ACEi, day3	hrACE2, day7	anti-IL6, day3	anti-iL6R, day3
Obesity	2.58	1.32	1.28	1.58	1.38	1.65	1.52	1.33	1.42	1.56
Heparin, day3			1.12	1.25	1.15	1.18	1.22	1.25	1.22	1.21
ICI, day3				1.16	1.06	1.21	1.19	1.26	1.15	1.17
Anti-viral, day3					1.18	1.55	1.48	1.2	1.25	1.22
Dex., day7						1.32	1.28	1.15	1.18	1.24
ARBs, day3							1.51	1.31	1.34	1.45
ACEi, day3								1.28	1.35	1.21
hrACE2, day7									1.15	1.18
anti-IL6, day3										1.11
anti-iL6R, day3										

Normalized Macrophages	Obesity	Heparin, day3	ICI, day3	Anti-viral, day3	Dex., day7	ARBs, day3	ACEi, day3	hrACE2, day7	anti-IL6, day3	anti-iL6R, day3
Obesity	5.35	2.25	1.88	2.62	1.62	2.35	2.75	1.92	1.88	1.72
Heparin, day3			1.38	1.78	1.32	2.11	2.23	1.65	1.71	1.75
ICI, day3				1.62	1.26	1.42	1.45	1.51	1.43	1.35
Anti-viral, day3					1.41	2.24	2.52	1.62	1.58	1.51
Dex., day7						1.35	1.41	1.31	1.28	1.36
ARBs, day3							2.24	1.84	1.62	1.56
ACEi, day3								1.58	1.56	1.44
hrACE2, day7									1.35	1.33
anti-IL6, day3										1.24
anti-iL6R, day3										

Favorable

Unfavorable

Table S4. Summary of model predictions for the therapeutic outcome of combined treatments for diabetic male patients (>65 years old). The table presents the results at the end of the simulation (day 20). Values have been normalized to the corresponding initial values except for S(pO2).

SpO_2 (%)	Diabetes	Heparin, day3	ICI, day3	Anti-viral, day3	Dex., day7	ARBs, day3	ACEi, day3	hrACE2, day7	anti-IL6, day3	anti-iL6R, day3
Diabetes	77	90	91	88	91	81	83	91	91	91
Heparin, day3			93	91	93	90	91	92	91	92
ICI, day3				92	94	91	91	93	93	93
Anti-viral, day3					92	90	90	91	92	92
Dex., day7						92	92	93	93	93
ARBs, day3							84	92	92	93
ACEi, day3								91	92	93
hrACE2, day7									93	93
anti-IL6, day3										93
anti-IL6R, day3										

Normalized T eff cells	Diabetes	Heparin, day3	ICI, day3	Anti-viral, day3	Dex., day7	ARBs, day3	ACEi, day3	hrACE2, day7	anti-IL6, day3	anti-iL6R, day3
Diabetes	1.02	1.21	1.38	1.15	1.42	1.15	1.13	1.32	1.22	1.28
Heparin, day3			1.45	1.26	1.51	1.25	1.23	1.41	1.32	1.35
ICI, day3				1.41	1.65	1.42	1.43	1.48	1.51	1.55
Anti-viral, day3					1.45	1.23	1.25	1.35	1.28	1.32
Dex., day7						1.48	1.45	1.52	1.57	1.61
ARBs, day3							1.18	1.35	1.28	1.33
ACEi, day3								1.38	1.25	1.31
hrACE2, day7									1.45	1.42
anti-IL6, day3										1.58
anti-iL6R, day3										

Lung Microthrombi	Diabetes	Heparin, day3	ICI, day3	Anti-viral, day3	Dex., day7	ARBs, day3	ACEi, day3	hrACE2, day7	anti-IL6, day3	anti-iL6R, day3
Diabetes	4.62	2.15	2.84	3.33	2.08	3.82	3.74	2.65	2.15	2.33
Heparin, day3			1.64	1.88	1.52	2.07	2.01	1.81	1.85	1.74
ICI, day3				2.52	1.81	2.64	2.38	1.64	1.58	1.64
Anti-viral, day3					2.01	3.15	3.05	2.25	2.08	2.15
Dex., day7						1.84	1.78	1.62	1.57	1.65
ARBs, day3							3.15	2.15	2.04	2.11
ACEi, day3								2.24	1.98	2.08
hrACE2, day7									1.64	1.74
anti-IL6, day3										1.62
anti-iL6R, day3										

Normalized IL6	Diabetes	Heparin, day3	ICI, day3	Anti-viral, day3	Dex., day7	ARBs, day3	ACEi, day3	hrACE2, day7	anti-IL6, day3	anti-iL6R, day3
Diabetes	5.20	2.64	2.78	3.84	2.52	3.74	3.65	2.25	2.14	2.08
Heparin, day3			1.74	2.44	2.15	2.64	2.73	1.95	1.64	1.71
ICI, day3				2.33	2.08	2.53	2.61	1.81	1.51	1.48
Anti-viral, day3					2.25	3.15	3.23	2.15	2.08	1.95
Dex., day7						1.92	2.14	1.88	1.92	1.75
ARBs, day3							3.15	2.01	1.94	1.81
ACEi, day3								2.15	2.01	1.95
hrACE2, day7									1.52	1.65
anti-IL6, day3										1.47
anti-iL6R, day3										

Inflammatory Cytokines	Diabetes	Heparin, day3	ICI, day3	Anti-viral, day3	Dex., day7	ARBs, day3	ACEi, day3	hrACE2, day7	anti-IL6, day3	anti-iL6R, day3
Diabetes	4.35	2.88	2.35	3.26	2.66	3.38	3.61	2.48	2.01	2.21
Heparin, day3			1.95	2.74	1.88	2.76	2.81	2.15	1.87	1.82
ICI, day3				2.15	1.52	2.31	2.33	1.94	1.72	1.65
Anti-viral, day3					2.43	3.15	3.08	2.15	1.83	1.91
Dex., day7						2.08	2.15	1.84	1.52	1.48
ARBs, day3							3.24	2.25	1.94	1.97
ACEi, day3								2.15	1.88	1.74
hrACE2, day7									1.45	1.49
anti-IL6, day3										1.38
anti-iL6R, day3										

Normalized Viral Load	Diabetes	Heparin, day3	ICI, day3	Anti-viral, day3	Dex., day7	ARBs, day3	ACEi, day3	hrACE2, day7	anti-IL6, day3	anti-iL6R, day3
Diabetes	6.88	4.58	2.45	1.88	2.15	3.15	3.22	2.88	2.25	2.52
Heparin, day3			2.08	1.52	1.84	2.98	3.08	2.15	1.94	1.81
ICI, day3				1.15	1.27	2.25	2.31	1.36	1.51	1.41
Anti-viral, day3					1.08	1.27	1.35	1.18	1.11	1.15
Dex., day7						1.35	1.41	1.13	1.08	1.13
ARBs, day3							2.52	2.28	2.34	2.41
ACEi, day3								2.35	2.42	2.54
hrACE2, day7									1.93	1.88
anti-IL6, day3										1.72
anti-iL6R, day3										

Normalized Neutrophils	Diabetes	Heparin, day3	ICI, day3	Anti-viral, day3	Dex., day7	ARBs, day3	ACEi, day3	hrACE2, day7	anti-IL6, day3	anti-iL6R, day3
Diabetes	2.72	1.88	1.71	2.15	1.72	1.98	2.15	1.65	1.71	1.62
Heparin, day3			1.58	1.95	1.38	1.93	2.08	1.51	1.45	1.38
ICI, day3				1.75	1.22	1.74	1.83	1.35	1.31	1.24
Anti-viral, day3					1.51	1.89	1.96	1.53	1.64	1.58
Dex., day7						1.64	1.68	1.44	1.48	1.31
ARBs, day3							2.08	1.62	1.68	1.56
ACEi, day3								1.57	1.63	1.53
hrACE2, day7									1.42	1.35
anti-IL6, day3										1.21
anti-iL6R, day3										

Normalized Macrophages	Diabetes	Heparin, day3	ICI, day3	Anti-viral, day3	Dex., day7	ARBs, day3	ACEi, day3	hrACE2, day7	anti-IL6, day3	anti-iL6R, day3
Diabetes	4.88	2.15	2.08	3.01	1.98	3.15	3.25	2.16	2.01	1.95
Heparin, day3			1.74	2.03	1.64	2.11	2.09	1.91	1.88	1.95
ICI, day3				1.84	1.52	1.86	1.93	1.75	1.61	1.57
Anti-viral, day3					1.71	1.94	1.96	1.83	1.73	1.68
Dex., day7						1.73	1.69	1.51	1.48	1.41
ARBs, day3							2.25	1.95	1.88	1.71
ACEi, day3								2.08	1.91	1.83
hrACE2, day7									1.56	1.51
anti-IL6, day3										1.48
anti-iL6R, day3										

Favorable

Unfavorable

Table S5. Summary of model predictions for the therapeutic outcome of combined treatments for hypertensive male patients (>65 years old). The table presents the results at the end of the simulation (day 20). Values have been normalized to the corresponding initial values except for S(pO2).

SpO_2 (%)	Hypertesion	Heparin, day3	ICI, day3	Anti-viral, day3	Dex., day7	ARBs, day3	ACEi, day3	hrACE2, day7	anti-IL6, day3	anti-iL6R, day3
Hypertesion	79	91	91	88	92	82	83	92	91	92
Heparin, day3			93	91	93	91	91	92	92	92
ICI, day3				93	94	92	92	93	93	93
Anti-viral, day3					92	90	90	92	93	92
Dex., day7						92	92	93	93	93
ARBs, day3							85	92	93	93
ACEi, day3								92	92	92.
hrACE2, day7									93	93
anti-IL6, day3										93
anti-iL6R, day3										

Normalized T eff cells	Hypertesion	Heparin, day3	ICI, day3	Anti-viral, day3	Dex., day7	ARBs, day3	ACEi, day3	hrACE2, day7	anti-IL6, day3	anti-iL6R, day3
Hypertesion	0.98	1.22	1.35	1.18	1.38	1.13	1.15	1.35	1.23	1.25
Heparin, day3			1.42	1.25	1.48	1.18	1.22	1.42	1.38	1.33
ICI, day3				1.38	1.58	1.38	1.41	1.51	1.48	1.52
Anti-viral, day3					1.41	1.25	1.23	1.38	1.26	1.38
Dex., day7						1.45	1.48	1.57	1.48	1.52
ARBs, day3							1.21	1.43	1.32	1.38
ACEi, day3								1.45	1.35	1.37
hrACE2, day7									1.42	1.45
anti-IL6, day3										1.51
anti-iL6R, day3										

Lung Microthrombi	Hypertesion	Heparin, day3	ICI, day3	Anti-viral, day3	Dex., day7	ARBs, day3	ACEi, day3	hrACE2, day7	anti-IL6, day3	anti-iL6R, day3
Hypertesion	4.34	1.88	2.14	3.54	1.98	3.67	3.81	2.32	2.17	2.25
Heparin, day3			1.54	1.81	1.48	1.85	1.87	1.78	1.71	1.81
ICI, day3				2.34	1.74	2.08	2.12	1.87	1.78	1.82
Anti-viral, day3					1.81	2.98	3.23	2.18	1.87	1.91
Dex., day7						1.78	1.81	1.87	1.69	1.71
ARBs, day3							3.17	2.18	1.91	2.13
ACEi, day3								2.22	2.03	2.11
hrACE2, day7									1.73	1.76
anti-IL6, day3										1.63
anti-iL6R, day3										

Normalized IL6	Hypertesion	Heparin, day3	ICI, day3	Anti-viral, day3	Dex., day7	ARBs, day3	ACEi, day3	hrACE2, day7	anti-IL6, day3	anti-iL6R, day3
Hypertesion	4.48	2.58	2.34	3.17	2.43	3.27	3.18	2.15	1.96	2.08
Heparin, day3			1.94	2.35	2.12	2.55	2.53	1.98	1.83	1.87
ICI, day3				2.28	2.08	2.29	2.23	1.82	1.48	1.55
Anti-viral, day3					2.23	3.08	2.96	2.01	1.92	1.96
Dex., day7						2.23	2.12	1.95	1.83	1.88
ARBs, day3							3.09	2.09	1.92	1.99
ACEi, day3								2.04	1.95	2.01
hrACE2, day7									1.77	1.81
anti-IL6, day3										1.45
anti-iL6R, day3										

Inflammatory Cytokines	Hypertesion	Heparin, day3	ICI, day3	Anti-viral, day3	Dex., day7	ARBs, day3	ACEi, day3	hrACE2, day7	anti-IL6, day3	anti-iL6R, day3
Hypertesion	4.00	2.78	2.25	3.14	2.29	3.26	3.48	2.45	2.16	2.11
Heparin, day3			2.02	2.67	1.74	2.61	2.69	1.98	1.86	1.92
ICI, day3				2.06	1.53	2.13	2.22	1.92	1.68	1.63
Anti-viral, day3					1.98	2.95	3.04	2.23	2.04	2.09
Dex., day7						1.95	2.01	1.74	1.62	1.58
ARBs, day3							3.11	2.24	2.09	2.03
ACEi, day3								2.18	1.99	1.85
hrACE2, day7									1.76	1.68
anti-IL6, day3										1.63
anti-iL6R, day3										

Normalized Viral Load	Hypertesion	Heparin, day3	ICI, day3	Anti-viral, day3	Dex., day7	ARBs, day3	ACEi, day3	hrACE2, day7	anti-IL6, day3	anti-iL6R, day3
Hypertesion	6.38	4.32	2.43	1.75	2.09	3.11	3.16	2.76	2.14	2.45
Heparin, day3			1.93	1.48	1.76	2.85	2.96	2.09	1.91	1.77
ICI, day3				1.12	1.27	2.14	2.22	1.28	1.43	1.38
Anti-viral, day3					1.03	1.16	1.23	1.15	1.08	1.11
Dex., day7						1.53	1.48	1.41	1.38	1.33
ARBs, day3							2.43	2.18	2.11	2.34
ACEi, day3								2.22	2.34	2.43
hrACE2, day7									1.87	1.75
anti-IL6, day3										1.64
anti-iL6R, day3										

Normalized Neutrophils	Hypertesion	Heparin, day3	ICI, day3	Anti-viral, day3	Dex., day7	ARBs, day3	ACEi, day3	hrACE2, day7	anti-IL6, day3	anti-iL6R, day3
Hypertesion	2.45	1.76	1.68	2.09	1.65	1.96	2.11	1.62	1.68	1.65
Heparin, day3			1.54	1.88	1.34	1.86	1.93	1.48	1.43	1.35
ICI, day3				1.68	1.18	1.67	1.76	1.28	1.31	1.28
Anti-viral, day3					1.45	1.83	1.88	1.46	1.53	1.55
Dex., day7						1.54	1.62	1.35	1.42	1.38
ARBs, day3							1.87	1.58	1.63	1.56
ACEi, day3								1.56	1.61	1.55
hrACE2, day7									1.37	1.35
anti-IL6, day3										1.32
anti-iL6R, day3										

Normalized Macrophages	Hypertesion	Heparin, day3	ICI, day3	Anti-viral, day3	Dex., day7	ARBs, day3	ACEi, day3	hrACE2, day7	anti-IL6, day3	anti-iL6R, day3
Hypertesion	5.28	2.21	2.13	3.22	2.15	3.28	3.32	2.22	2.18	2.11
Heparin, day3			1.83	2.11	1.68	2.23	2.14	1.96	1.91	1.99
ICI, day3				1.87	1.56	1.87	1.95	1.77	1.64	1.62
Anti-viral, day3					1.74	2.03	2.11	1.93	1.82	1.77
Dex., day7						1.75	1.71	1.58	1.55	1.44
ARBs, day3							2.33	2.04	1.96	1.87
ACEi, day3								2.11	1.98	1.85
hrACE2, day7									1.65	1.61
anti-IL6, day3										1.55
anti-iL6R, day3										

Favorable

Unfavorable

Table S6. Summary of model predictions for the therapeutic outcome of combined treatments for hyper-inflamed male patients (>65 years old). The table presents the results at the end of the simulation (day 20). Values have been normalized to the corresponding initial values except for S(pO2).

SpO_2 (%)	Hyper inflamed	Heparin, day3	ICI, day3	Anti-viral, day3	Dex., day7	ARBs, day3	ACEi, day3	hrACE2, day7	anti-IL6, day3	anti-iL6R, day3
Hyper inflamed	76	89	91	89	91	82	83	87	91	91
Heparin, day3			92	90	92	89	89	90	91	91
ICI, day3				92	93	92	92	92	93	94
Anti-viral, day3					92	90	91	90	92	93
Dex., day7						92	92	92	93	93
ARBs, day3							85	88	91	91
ACEi, day3								89	91	91
hrACE2, day7									93	94
anti-IL6, day3										94
anti-iL6R, day3										

Normalized T eff cells	Hyper inflamed	Heparin, day3	ICI, day3	Anti-viral, day3	Dex., day7	ARBs, day3	ACEi, day3	hrACE2, day7	anti-IL6, day3	anti-iL6R, day3
Hyper inflamed	0.95	1.15	1.35	1.12	1.34	1.08	1.11	1.21	1.15	1.24
Heparin, day3			1.42	1.22	1.35	1.28	1.26	1.28	1.32	1.34
ICI, day3				1.42	1.58	1.38	1.41	1.48	1.56	1.62
Anti-viral, day3					1.38	1.15	1.21	1.32	1.25	1.31
Dex., day7						1.18	1.22	1.41	1.51	1.54
ARBs, day3							1.25	1.31	1.42	1.38
ACEi, day3								1.35	1.37	1.41
hrACE2, day7									1.47	1.52
anti-IL6, day3										1.62
anti-iL6R, day3										

Lung Microthrombi	Hyper inflamed	Heparin, day3	ICI, day3	Anti-viral, day3	Dex., day7	ARBs, day3	ACEi, day3	hrACE2, day7	anti-IL6, day3	anti-iL6R, day3
Hyper inflamed	6.02	2.31	2.45	3.15	2.15	3.54	3.67	2.54	2.08	1.95
Heparin, day3			1.74	1.88	1.71	1.78	1.82	1.65	1.58	1.52
ICI, day3				1.78	1.82	2.15	2.22	1.88	1.42	1.45
Anti-viral, day3					1.98	2.75	2.52	1.94	1.74	1.67
Dex., day7						1.89	1.75	1.62	1.55	1.68
ARBs, day3							3.24	2.41	1.95	1.84
ACEi, day3								2.34	1.88	1.71
hrACE2, day7									1.78	1.62
anti-IL6, day3										1.58
anti-iL6R, day3										

Normalized IL6	Hyper inflamed	Heparin, day3	ICI, day3	Anti-viral, day3	Dex., day7	ARBs, day3	ACEi, day3	hrACE2, day7	anti-IL6, day3	anti-iL6R, day3
Hyper inflamed	5.98	2.74	2.65	3.25	2.45	3.28	3.15	2.25	1.98	2.08
Heparin, day3			1.88	2.55	2.34	2.61	2.56	2.08	1.54	1.62
ICI, day3				2.35	2.22	2.58	2.41	1.78	1.32	1.35
Anti-viral, day3					1.88	2.35	2.14	1.84	1.62	1.75
Dex., day7						2.08	1.98	1.62	1.58	1.56
ARBs, day3							3.07	2.02	1.74	1.81
ACEi, day3								2.15	1.81	1.92
hrACE2, day7									1.34	1.39
anti-IL6, day3										1.28
anti-iL6R, day3										

Inflammatory Cytokines	Hyper inflamed	Heparin, day3	ICI, day3	Anti-viral, day3	Dex., day7	ARBs, day3	ACEi, day3	hrACE2, day7	anti-IL6, day3	anti-iL6R, day3
Hyper inflamed	4.62	3.15	2.88	3.18	2.54	3.52	3.64	2.52	2.05	2.15
Heparin, day3			2.15	2.94	2.12	2.95	3.06	2.32	1.54	1.62
ICI, day3				2.59	1.94	2.75	2.81	2.15	1.41	1.38
Anti-viral, day3					2.45	3.02	2.96	2.46	1.78	1.98
Dex., day7						2.25	2.08	1.64	1.22	1.25
ARBs, day3							2.15	1.85	1.78	1.75
ACEi, day3								1.92	1.81	1.86
hrACE2, day7									1.35	1.42
anti-IL6, day3										1.22
anti-iL6R, day3										

Normalized Viral Load	Hyper inflamed	Heparin, day3	ICI, day3	Anti-viral, day3	Dex., day7	ARBs, day3	ACEi, day3	hrACE2, day7	anti-IL6, day3	anti-iL6R, day3
Hyper inflamed	1.42	1.38	1.32	1.12	1.35	1.40	1.38	1.35	1.22	1.18
Heparin, day3			1.28	1.08	1.23	1.38	1.34	1.32	1.21	1.15
ICI, day3				1.02	1.16	1.25	1.28	1.2	1.01	1.05
Anti-viral, day3					1.05	1.08	1.05	1.05	1.02	1.02
Dex., day7						1.31	1.3	1.28	1.22	1.15
ARBs, day3							1.35	1.31	1.21	1.18
ACEi, day3								1.33	1.18	1.15
hrACE2, day7									1.18	1.14
anti-IL6, day3										1.11
anti-iL6R, day3										

Normalized Neutrophils	Hyper inflamed	Heparin, day3	ICI, day3	Anti-viral, day3	Dex., day7	ARBs, day3	ACEi, day3	hrACE2, day7	anti-IL6, day3	anti-iL6R, day3
Hyper inflamed	2.88	1.95	1.88	2.15	1.86	2.22	2.35	1.78	1.62	1.75
Heparin, day3			1.75	1.92	1.56	2.08	2.15	1.65	1.58	1.45
ICI, day3				1.65	1.38	1.85	1.79	1.52	1.45	1.35
Anti-viral, day3					1.45	1.95	2.08	1.72	1.59	1.63
Dex., day7						1.75	1.66	1.52	1.22	1.25
ARBs, day3							2.15	1.64	1.53	1.41
ACEi, day3								1.62	1.55	1.65
hrACE2, day7									1.31	1.25
anti-IL6, day3										1.15
anti-iL6R, day3										

Normalized Macrophages	Hyper inflamed	Heparin, day3	ICI, day3	Anti-viral, day3	Dex., day7	ARBs, day3	ACEi, day3	hrACE2, day7	anti-IL6, day3	anti-iL6R, day3
Hyper inflamed	5.48	3.15	2.98	3.25	3.15	3.22	2.75	2.58	2.25	2.42
Heparin, day3			2.62	2.96	2.14	3.15	2.65	2.35	1.95	1.72
ICI, day3				2.56	1.52	2.57	2.35	1.72	1.62	1.42
Anti-viral, day3					1.62	2.74	2.68	2.22	1.88	1.68
Dex., day7						1.71	1.68	1.54	1.45	1.52
ARBs, day3							2.56	2.14	1.96	1.88
ACEi, day3								2.25	1.78	1.82
hrACE2, day7									1.45	1.36
anti-IL6, day3										1.32
anti-iL6R, day3										

Favorable

Unfavorable

Table S7. Model predictions for the treatment of an at-risk (age>65, obese, hypertensive, diabetic and hyper-inflamed) male patient using additional spike of interferon, initiated at day 3 or 7.

Baseline	Age>65	Obesity	Hypertesion	Diabetes	Hyper inflamed
SpO_2 (%)	82	80	79	78	76
Microthrombosis in lung	5.58	4.98	4.34	4.62	6.02
Cytokines cells	3.87	4.15	4.00	4.35	4.62
T eff cells	1.00	1.12	0.98	1.02	0.95
IL-6	4.68	5.54	4.48	5.20	5.98
Viral Load	7.45	5.98	6.38	6.88	1.42
Neutrophils	2.30	2.58	2.45	2.72	2.88
Macrophages	5.15	5.35	5.28	4.88	5.48

Interferon day3	Age>65	Obesity	Hypertesion	Diabetes	Hyper inflamed
SpO_2 (%)	84	83	85	84	83
Microthrombosis in lung	3.56	3.15	3.33	3.65	3.74
Cytokines cells	2.12	2.25	2.41	3.25	3.08
T eff cells	1.25	1.05	1.15	1.18	1.16
IL-6	3.15	3.64	3.52	3.65	3.45
Viral Load	4.15	3.98	3.65	4.08	1.25
Neutrophils	1.23	1.85	1.75	2.08	1.84
Macrophages	3.15	3.35	3.12	3.45	3.84

Interferon day7	Age>65	Obesity	Hypertesion	Diabetes	Hyper inflamed
SpO_2 (%)	83	82	84	83	82
Microthrombosis in lung	3.78	3.23	3.42	3.73	3.82
Cytokines cells	2.23	2.33	2.53	3.33	3.19
T eff cells	1.08	1.02	1.04	1.09	1.05
IL-6	3.23	3.76	3.63	3.75	3.56
Viral Load	4.56	4.12	3.78	4.18	1.36
Neutrophils	1.35	2.02	1.87	2.25	1.96
Macrophages	3.24	3.45	3.26	3.63	3.98

SI References

1. S. W. Friedrich *et al.*, Antibody-directed effector cell therapy of tumors: analysis and optimization using a physiologically based pharmacokinetic model. *Neoplasia* **4**, 449-463 (2002).
2. R. J. Melder *et al.*, Systemic distribution and tumor localization of adoptively transferred lymphocytes in mice: comparison with physiologically based pharmacokinetic model. *Neoplasia* **4**, 3-8 (2002).
3. L. T. Baxter, R. K. Jain, Pharmacokinetic analysis of the microscopic distribution of enzyme-conjugated antibodies and prodrugs: comparison with experimental data. *Br J Cancer* **73**, 447-456 (1996).
4. L. T. Baxter, H. Zhu, D. G. Mackensen, W. F. Butler, R. K. Jain, Biodistribution of monoclonal antibodies: scale-up from mouse to human using a physiologically based pharmacokinetic model. *Cancer Res* **55**, 4611-4622 (1995).
5. L. T. Baxter, H. Zhu, D. G. Mackensen, R. K. Jain, Physiologically based pharmacokinetic model for specific and nonspecific monoclonal antibodies and fragments in normal tissues and human tumor xenografts in nude mice. *Cancer Res* **54**, 1517-1528 (1994).
6. M. R. Pilvankar, H. L. Yong, A. N. Ford Versypt, A Glucose-Dependent Pharmacokinetic/Pharmacodynamic Model of ACE Inhibition in Kidney Cells. *Processes* **7**, 131 (2019).
7. M. R. Pilvankar, M. A. Higgins, A. N. F. Versypt, Mathematical Model for Glucose Dependence of the Local Renin–Angiotensin System in Podocytes. *Bulletin of mathematical biology* **80**, 880-905 (2018).
8. A. Lo *et al.*, "Using a systems biology approach to explore hypotheses underlying clinical diversity of the renin angiotensin system and the response to antihypertensive therapies" in Clinical trial simulations. (Springer, 2011), pp. 457-482.
9. K. J. Mahasa, A. Eladdadi, L. De Pillis, R. Ouifki, Oncolytic potency and reduced virus tumor-specificity in oncolytic virotherapy. A mathematical modelling approach. *Plos one* **12**, e0184347 (2017).
10. J. L. Dunster, H. M. Byrne, J. R. King, The resolution of inflammation: a mathematical model of neutrophil and macrophage interactions. *Bulletin of mathematical biology* **76**, 1953-1980 (2014).
11. E. R. Weibel, B. Sapoval, M. Filoche, Design of peripheral airways for efficient gas exchange. *Respiratory physiology & neurobiology* **148**, 3-21 (2005).
12. X. Lai, A. Friedman, Combination therapy of cancer with cancer vaccine and immune checkpoint inhibitors: A mathematical model. *PLoS One* **12**, e0178479 (2017).
13. S. Baral, R. Antia, N. M. Dixit, A dynamical motif comprising the interactions between antigens and CD8 T cells may underlie the outcomes of viral infections. *Proceedings of the National Academy of Sciences* **116**, 17393-17398 (2019).
14. W. Mok, T. Stylianopoulos, Y. Boucher, R. K. Jain, Mathematical modeling of herpes simplex virus distribution in solid tumors: implications for cancer gene therapy. *Clin Cancer Res* **15**, 2352-2360 (2009).
15. A. M. Smith, J. A. McCullers, F. R. Adler, Mathematical model of a three-stage innate immune response to a pneumococcal lung infection. *Journal of theoretical biology* **276**, 106-116 (2011).
16. Z. Su, Y. Wu, A multiscale and comparative model for receptor binding of 2019 novel coronavirus and the implication of its life cycle in host cells. *BioRxiv* (2020).
17. F. Mpekris, S. Angeli, A. P. Pirentis, T. Stylianopoulos, Stress-mediated progression of solid tumors: effect of mechanical stress on tissue oxygenation, cancer cell proliferation, and drug delivery. *Biomech Model Mechanobiol* **14**, 1391-1402 (2015).

18. T. K. Roy, T. W. Secomb, Theoretical analysis of the determinants of lung oxygen diffusing capacity. *J Theor Biol* **351**, 1-8 (2014).
19. H. Zhu, R. J. Melder, L. T. Baxter, R. K. Jain, Physiologically based kinetic model of effector cell biodistribution in mammals: implications for adoptive immunotherapy. *Cancer Res* **56**, 3771-3781 (1996).
